# Discovery, characterization, and structure of a cofactor-independent histidine racemase from the oral pathogen *Fusobacterium nucleatum*

**DOI:** 10.1016/j.jbc.2024.107896

**Published:** 2024-10-17

**Authors:** Tess Lamer, Pu Chen, Marie J. Venter, Marco J. van Belkum, Anjalee Wijewardane, Chenggang Wu, M. Joanne Lemieux, John C. Vederas

**Affiliations:** 1 Department of Chemistry, University of Alberta, Edmonton, Alberta, Canada; 2 Department of Biochemistry, University of Alberta, Edmonton, Alberta, Canada; 3 Li Ka Shing Institute of Virology, University of Alberta, Edmonton, Alberta, Canada; 4 Department of Microbiology and Molecular Genetics, University of Texas McGovern Medical School, Houston, Texas, USA

**Keywords:** D-histidine, racemase, stereochemistry, cofactor-independent, lanthionine, *Fusobacterium nucleatum*, staphylopine, CntK, *Staphylococcus aureus*

## Abstract

*Fusobacterium nucleatum* is an oral commensal bacterium that can act as an opportunistic pathogen and is implicated in diseases such as periodontitis, adverse pregnancy outcomes, colorectal cancer, and Alzheimer’s disease. *F. nucleatum* synthesizes lanthionine for its peptidoglycan, rather than *meso*-2,6-diaminopimelic acid (DAP) used by most Gram-negative bacteria. Despite lacking the biosynthetic pathway for DAP, the genome of *F. nucleatum* ATCC 25586 encodes a predicted DAP epimerase. A recent study hypothesized that this enzyme may act as a lanthionine epimerase, but the authors found a very low turnover rate, suggesting that this enzyme likely has another more favored substrate. Here, we characterize this enzyme as a histidine racemase (HisR), and found that catalytic turnover is ∼10,000× faster with L-histidine than with L,L-lanthionine. Kinetic experiments suggest that HisR functions as a cofactor-independent racemase and that turnover is specific for histidine, while crystal structures of catalytic cysteine to serine mutants (C67S or C209S) reveal this enzyme in its substrate-unbound, open conformation. Currently, the only other reported cofactor-independent histidine racemase is CntK from *Staphylococcus aureus*, which is used in the biosynthesis of staphylopine, a broad-spectrum metallophore that increases virulence of *S. aureus*. However, CntK shares only 28% sequence identity with HisR, and their genes exist in different genomic contexts. Knockout of *hisR* in *F. nucleatum* results in a small but reproducible lag in growth compared to WT during exponential phase, suggesting that HisR may play a role in growth of this periodontal pathogen.

*Fusobacterium nucleatum* is a Gram-negative, obligate anaerobe bacterium found in the human oral microbiome (1). Although typically a commensal organism, *F. nucleatum* can act as an opportunistic pathogen, and is implicated in a wide variety of human diseases including but not limited to colorectal cancer, adverse pregnancy outcomes, periodontitis, and Alzheimer’s disease (2, 3). In fact, of bacteria associated with periodontal disease, *F. nucleatum* is the most common organism found in clinical infections of other body sites (4, 5). Of particular interest is this organism’s role in colorectal cancer. Extensive research in this area has shown that *F. nucleatum* can drive progression of colorectal cancer through induction of the host inflammatory response, resulting in creation of a microenvironment that favors tumor growth, as well as by inhibiting the action of human immune cells and promoting resistance to chemotherapy (6, 7, 8, 9).

Interestingly, *F. nucleatum* lacks the biosynthetic pathway to produce *meso*-2,6-diaminopimelic acid (D,L-DAP), which is commonly used by Gram-negative bacteria as the crosslinker between peptidoglycan pentapeptide chains (10). Instead, this organism synthesizes and incorporates D,L-lanthionine into its peptidoglycan (11, 12). The biosynthesis of lanthionine in *F. nucleatum* is completed by the pyridoxal 5′-phosphate (PLP)-dependent enzyme lanthionine synthase (13, 14). The reaction produces hydrogen sulfide as a byproduct and is thought to contribute to malodor exhibited by patients with oral *F. nucleatum* infections (15). A recent detailed biochemical study by Darbyshire *et al.* found that lanthionine synthase produces L,L-lanthionine by condensation of two molecules of L-cysteine, and D,L-lanthionine by the condensation of L-cysteine with D-cysteine (16). In the presence of equimolar concentrations of cysteine enantiomers, lanthionine synthase favored formation of D,L- over L,L-lanthionine 3:1.

While it is possible that *F. nucleatum* can synthesize or access D-cysteine from the environment (17, 18, 19), the genome of *F. nucleatum* American Type Culture Collection (ATCC) 25586 encodes a predicted DAP epimerase enzyme. DAP epimerase catalyzes the reversible conversion of L,L-DAP to D,L-DAP and is the second last step in lysine biosynthesis in many bacterial species (20). DAP epimerase is a cofactor-independent enzyme (also referred to as pyridoxal 5′-phosphate-independent), and remarkably can invert the stereochemistry of an amino acid α-H (p*K*_a_ ∼ 29) using a pair of catalytic cysteine residues in its active site, which exist as a thiol-thiolate pair (p*K*_a_ ∼ 9) (21). Since *F. nucleatum* lacks the ability to produce DAP and lysine, Darbyshire *et al.* hypothesized that this enzyme may actually function to produce D,L-lanthionine from L,L-lanthionine, providing the last step in the biosynthetic pathway of lanthionine in this organism (16). However, they found a very low turnover rate with lanthionine (0.07 s^−1^), and the authors concluded that this enzyme likely has another more favored substrate.

In this work, we set out to find the physiological substrate of the cofactor-independent racemase (predicted DapF, FN1732) from *F. nucleatum* ATCC 25586, and were surprised to find that this enzyme functions as a histidine racemase (HisR). We characterized the kinetics of this enzyme with D- and L-histidine, and solved two crystal structures of active site mutants (C67S and C209S). The structures obtained are very similar to the crystal structure of the only other characterized cofactor-independent histidine racemase, CntK from *Staphylococcus aureus* Mu50/ATCC 700699 (22). Genetic comparison of *F. nucleatum* and *S. aureus* suggests that these two enzymes have different roles in their respective organisms (23, 24, 25). Genetic knockout of *hisR* in a closely related strain of *F. nucleatum* (ATCC 23726) resulted in a small but reproducible lag in growth during exponential phase, suggesting a role for HisR in growth of this periodontal pathogen.

## Results

### Enzyme substrate identification and scope

To determine the substrate of FN1732 from *F. nucleatum* ATCC 25586, we first used several different protein function prediction tools (Table S1). A simple pBLAST search predicted this enzyme as a diaminopimelic acid epimerase (DapF) based on the amino acid sequence, while functional predictions based on protein structural models (Phyre2 or AlphaFold2 with FoldSeek) both annotated this enzyme as a histidine racemase by identifying CntK from *S. aureus* as the top structural hit (22, 26, 27).

**Figure 1:**
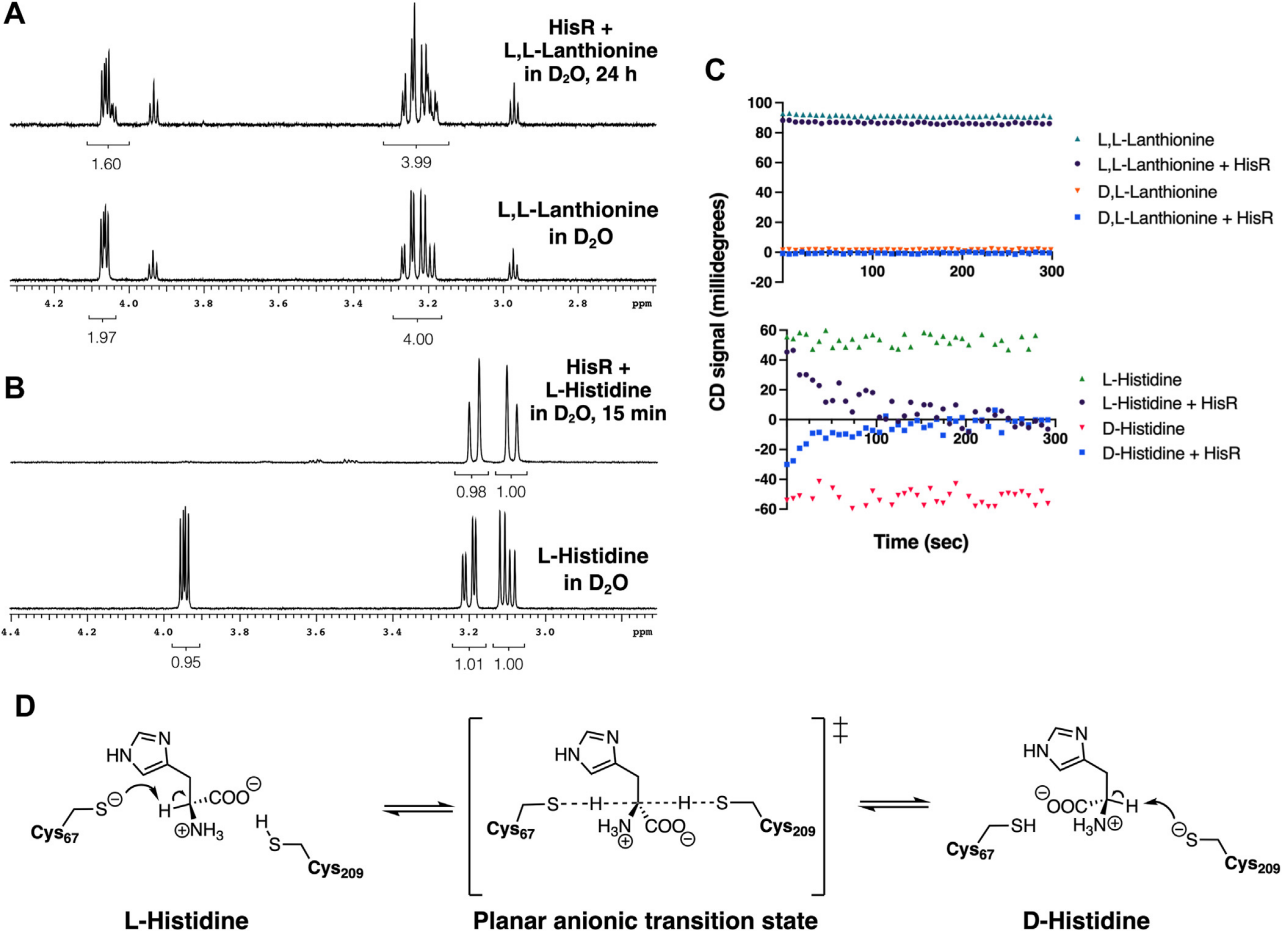
***Fusobacterium nucleatum* ATCC 25586 DapF is a histidine racemase enzyme, and substrate turnover is faster with histidine than with lanthionine.***A*, D_2_O ^1^H-NMR assay of *F. nucleatum* ATCC 25586 HisR with L,L-lanthionine. Solutions of 5 mM L,L-lanthionine in D_2_O buffer were prepared and analyzed with a 600 MHz NMR spectrometer. ∼10 μg of HisR enzyme diluted in D_2_O buffer was added, and spectra were collected again after 24 h. The small triplets at 3.94 and 2.98 ppm are from 2-mercaptoethanol, which was added to experiments longer than 8 h to keep HisR reduced. *B*, D_2_O ^1^H-NMR assay of *F. nucleatum* ATCC 25586 HisR with L-histidine. Solutions of 5 mM L-histidine in D_2_O buffer were prepared and analyzed with a 600 MHz NMR spectrometer. ∼10 μg of HisR enzyme diluted in D_2_O buffer was added, and spectra were collected again after 15 min. *C*, circular dichroism monitoring of HisR enzyme (5 μg) activity with 30 mM solutions of either lanthionine or histidine. The CD signal was recorded at 212 nm, and the average integrated CD signal in mdeg was recorded every five seconds. Experiments were performed in duplicate and CD signals displayed are the average between the two trials. *D*, proposed enzyme mechanism of *F. nucleatum* HisR, based on the general mechanism of cofactor-independent (PLP-independent) racemase enzymes. ATCC, American Type Culture Collection; DapF, diaminopimelic acid epimerase; HisR, histidine racemase; PLP, pyridoxal 5′-phosphate.

With this in mind, FN1732 was expressed in *Escherichia coli* with a C-terminal hexahistidine tag and purified. The enzyme was highly soluble after overexpression in *E. coli*, and typical yields were >15 mg/L. We then proceeded to screen amino acid substrates for this enzyme using a D_2_O ^1^H-NMR assay (28). In this experiment, enzyme is added to a solution of a potential substrate in a D_2_O buffer. If the amino acid is indeed a substrate for the racemase or epimerase enzyme, the ^1^H-NMR signal for the α-H will disappear as it is replaced with the ^1^H-NMR inactive isotope deuterium, and the splitting patterns of any β-H signals will simplify. We screened all 20 proteinogenic amino acids, in addition to several other substrates, for a total of 35 compounds (Fig. S1). After 24 to 48 h, ^1^H-NMR spectra were obtained and analyzed for loss of the α-H signal and changes in coupling patterns of the β-H. For lanthionine, ∼20% loss of signal was observed after 24 h, along with appearance of new signals for the β-H as replacement of one α-H with deuterium generates an (α-D)-D,L-lanthionine molecule, where all four β-H produce separate signals appearing as either doublets or doublets of doublets (Fig. 1*A*). This demonstrated that lanthionine could act as a substrate for FN1732, albeit at a very slow rate, agreeing with the results of Darbyshire *et al.* (16).

In contrast, we found that after only 15 min of incubation with the same concentration of FN1732, the α-H signal of L-histidine completely disappeared and both β-H signals simplified to doublets, suggesting that histidine was the true substrate for FN1732 (Fig. 1*B*). This result was confirmed using circular dichroism (CD). Upon incubation of 30 mM solutions of either lanthionine or histidine with FN1732, noticeable change in the CD signal of lanthionine was not observed, while histidine solutions of either enantiomer with the same concentration of enzyme were converted into racemic mixtures in ∼300 s (Fig. 1*C*). Aside from histidine and lanthionine, none of the other substrates tested displayed any turnover with HisR in the D_2_O ^1^H-NMR assay. Taken together, these results suggest histidine as the true substrate for this racemase FN1732, which we refer to now as HisR. A proposal for the mechanism of HisR is shown in Figure 1*D*, based on the conserved mechanism for this class of enzymes (21).

We also explored substrate specificity with analogues of histidine in the same assay (Fig. S2). Alteration of either the backbone atoms or side chain of histidine prevented any of these compounds from being used as substrates by HisR. Notably, we synthesized an oxazole analogue of histidine, which surprisingly was not tolerated as a substrate by HisR after 24 h, suggesting a high degree of catalytic specificity for histidine by this enzyme.

### Kinetic studies of HisR with histidine

The kinetics of HisR with histidine as a substrate were determined using CD spectroscopy. As expected, the *k*_cat_ value was approximately 10,000× greater for L-histidine than L,L-lanthionine, and nearly 40,000× greater with D-histidine than with L,L-lanthionine (Table 1 and Fig. S3). Interestingly, the *K*_M_ value of HisR was lower with L,L-lanthionine than with either enantiomer of histidine. While a *K*_M_ in the mM range may seem relatively high, this is typical for cofactor-independent amino acid racemases (29, 30, 31), apart from DAP epimerase and glutamate racemase, which have *K*_M_ values in the μM range (32, 33, 34).

**Table 1:** Kinetic parameters of *F. nucleatum* ATCC 25586 HisR with histidine or lanthionine as a substrate

Substrate	*K*_M_ (mM)	*k*_cat_ (s^−1^)	*k*_cat_/*K*_M_ (M^−1^·s^−1^)
L-Histidine	4	74	2 × 10^4^
D-Histidine	24	250	1 × 10^4^
L,L-Lanthionine^a^	1.9	0.007	38

a Determined by Darbyshire *et al.* (16).

### HisR enzyme mechanism

**Figure 2:**
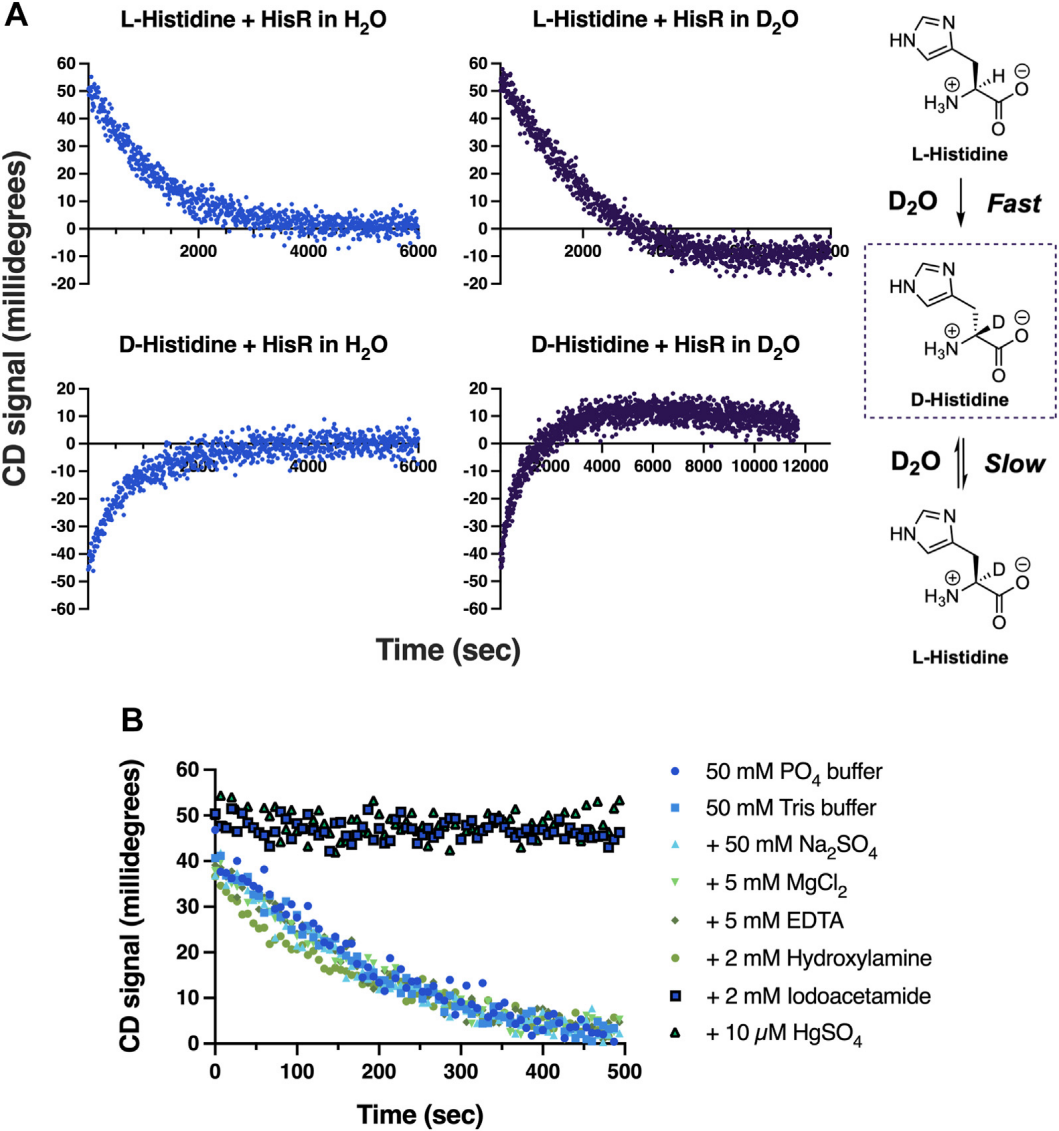
***Fusobacterium nucleatum* ATCC 25586 histidine racemase operates *via* a two-base mechanism.***A*, circular dichroism of HisR with histidine in a D_2_O buffer shows a single overshoot, suggesting a two base mechanism. 30 mM solutions of L- or D-histidine in H_2_O or D_2_O buffer were prepared, and then HisR (0.5 μg in H_2_O and 5 μg in D_2_O) was added. The CD signal was recorded at 212 nm, and the average integrated CD signal in mdeg was recorded every five seconds. Experiments were performed in duplicate and CD signals displayed are the average between the two trials. The reaction scheme on the right offers an explanation of the single overshoot observed when L-histidine is added to HisR in D_2_O buffer. The initial racemization reaction proceeds rapidly to produce α-D D-histidine, where the deuterium originates from buffer exchange. The reverse reaction, racemization of α-D D-histidine to α-D L-histidine, proceeds more slowly due to the kinetic isotope effect. Therefore, the system has not yet reached isotopic equilibrium at the initial point of stereoisomeric equilibrium, resulting in the CD signal overshoot observed in D_2_O but not in H_2_O. *B*, enzyme activity in the presence of potential inhibitors and other additives. All experiments were run in 50 mM PO_4_, 100 mM NaCl, pH 8, except for the Tris buffer condition, which was run in 50 mM Tris, 100 mM NaCl, pH 8. A 300 mM (10×) solution of L-histidine was prepared and then added to an enzyme solution (3 μg) that had been preincubated with the appropriate additive for 1 h. The CD signal was recorded at 212 nm, and the average integrated CD signal in mdeg was recorded every five seconds. Experiments were performed in duplicate and CD signals displayed are the average between the two trials. ATCC, American Type Culture Collection; HisR, histidine racemase.

To confirm that HisR functions as a cofactor-independent racemase, we conducted a series of classical CD experiments. The observation of a D_2_O “overshoot” of cofactor-independent racemase and epimerases was first observed in 1968 with proline racemase (31), and later with glutamate racemase, DAP epimerase, and *O*-ureidoserine racemase (35, 36, 37). In the presence of D_2_O, cofactor-independent racemases will initially remove the α-H of their amino acid substrate and replace it with a solvent-derived deuterium atom using a two-base mechanism that relies on catalytic cysteine residues forming a thiol-thiolate pair. This initial reaction proceeds rapidly to produce the substrate enantiomer (or diastereomer) as a product. The subsequent reverse stereoisomerization reaction now proceeds more slowly due to the kinetic isotope effect. As a result, the initial point of stereoisomeric equilibrium is “overshot”, and an overabundance of the initial deuterated product is produced as the reaction reaches isotopic equilibrium (Fig. 2*A*). Eventually the reaction will reach stereoisomeric equilibrium, allowing the “overshoot” to be observed with CD. This mechanism is characteristic for cofactor-independent racemase and epimerase enzymes and was clearly observed with HisR.

**Figure 3:**
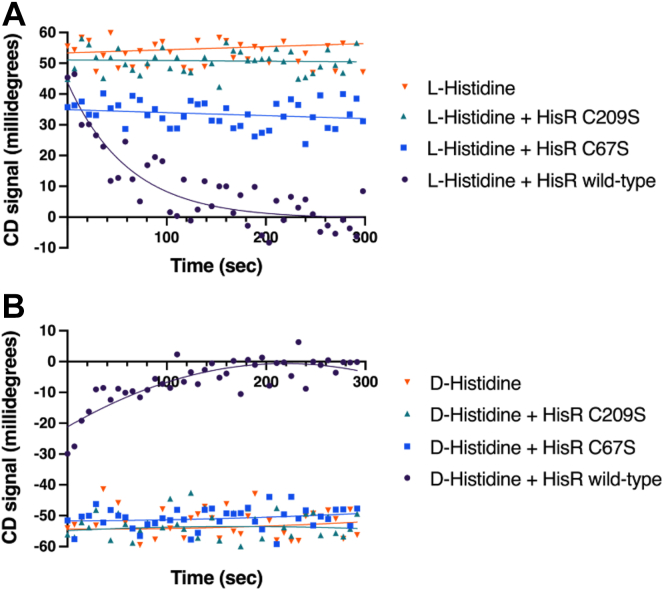
**Relative enzyme activity of *Fusobacterium nucleatum* ATCC 25586 HisR active site mutants compared to the WT enzyme.***A*, circular dichroism of HisR (67 μg/ml) or its mutants with L-histidine (30 mM). *B*, circular dichroism of HisR (67 μg/ml) or its mutants with D-histidine (30 mM). ATCC, American Type Culture Collection; HisR, histidine racemase.

We further confirmed this enzyme as a cofactor-independent racemase through a series of CD experiments in the presence of various buffer additives and potential inhibitors of enzyme activity (Fig. 2*B*). The rate of HisR was not affected by buffer composition (PO_4_ or Tris), ions (SO_4_^2−^, Mg^2+^, and EDTA), or the PLP inhibitor hydroxylamine; however, the enzyme activity was hindered by iodoacetamide and Hg^2+^, which are known inhibitors of enzymes with catalytic thiols. These results, along with a sequence alignment of HisR with other cofactor-independent racemase and epimerase enzymes, suggested that HisR operates *via* a two-base mechanism using cysteine residues 67 and 209. To confirm this, we used site-directed mutagenesis to generate HisR mutants C67S and C209S. Catalytic turnover of these mutants was greatly reduced with D- and L-histidine (Fig. 3) compared to the WT enzyme, as expected (38).

### Crystal structures of C67S and C209S mutants of HisR

Mutation of an active site cysteine to serine is a common strategy to aid crystallization of cofactor-independent epimerases and racemases, and likely aids the process because this mutation slows catalysis and helps to keep the enzyme in the open conformation (39). The crystal structures of HisR mutants C67S (Protein Data Bank (PDB) 9CR1) and C209S (PDB 9CR6) were each solved to a resolution of 2.5 Å (Table 2), containing four chains in both mutants. In the asymmetric unit of C67S, there are two back-to-back dimers, while the asymmetric unit of C209S has one dimer and two monomers. The electron density fits the active site residues of both mutants well (Fig. S5, *A* and *B*).

**Table 2:** Data collection and refinement statistics^a^

	HisR C67S	HisR C209S
Data collection	9CR1	9CR6
Space group	C 1 2 1	P 1 2_1_ 1
Cell dimensions		
a, b, c (Å)	72.14 149.61 138.23	72.85 89.12 100.33
α, β, γ (°)	90.00 89.98 90.00	90.00 100.91 90.00
Resolution (Å)	36.10–2.50	35.76–2.49
*R*_merge_ (last shell)	0.10 (1.16)	0.189 (2.121)
*I*/σ*I* (last shell)	9.94 (1.16)	5.44 (0.96)
Completeness % (last shell)	97.35 (94.29)	97.86 (97.06)
Observations (last shell)	348270 (33817)	295769 (29039)
Redundancy	7.1	6.8
Refinement		
Resolution Å	2.59–2.50	2.58–2.49
No. reflections (No. reflections in cross validation)	49178 (4758)	43343 (4254)
*R*_work_/*R*_free_ (last shell)	0.25/0.30 (0.52/0.52)	0.26/0.31 (0.41/0.46)
No. atoms	8564	8260
Protein	8450	8223
Ligand/ion	40	15
Water	74	22
*B*-factors	94.25	72.30
Protein	94.69	72.25
Ligand/ion	101.26	103.43
Water	40.50	72.30
R.m.s. deviations		
Bond lengths (Å)	0.004	0.003
Bond angles (°)	0.61	0.53

a Statistics for the highest-resolution shell are shown in parentheses.

**Figure 4:**
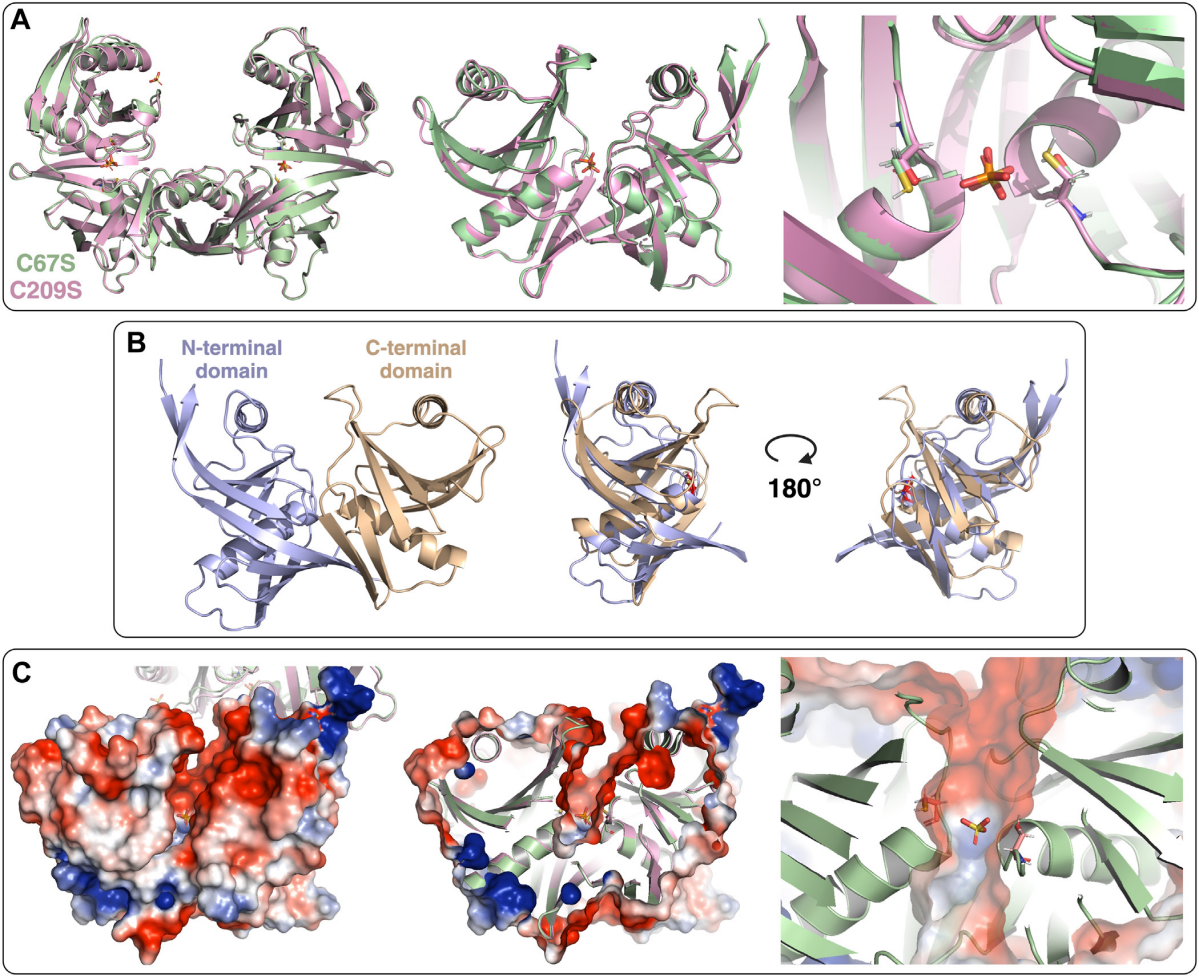
**Crystal structures of C67S (PDB****9CR1****) and C209S (PDB****9CR6****) HisR mutant enzymes from *Fusobacterium nucleatum* ATCC 25586 at 2.5 Å.***A*, overlay of C67S (*green*) and C209S (*pink*) HisR crystal structures, with backbone r.m.s.d. of 0.493 Å by aligning α-carbons of 260 residues. The *left panel* shows an overlay of homodimers formed by HisR C67S and C209S, with the dimerization interface shown in the *middle* of the structure. The *middle panel* shows an overlay of monomer units of C67S and C209S HisR enzymes. The active site of each enzyme is occupied by a sulfate (C67S) or phosphate (C209S) ion. The *right panel* shows an overlay of active site residues of C67S and C209S HisR enzymes, with catalytic cysteines/serines indicated. *B*, N-terminal (*purple*) and C-terminal (*orange*) domains of HisR. The *left panel* shows a color-coded representation of the two domains, while the *right panels* show a structural alignment of the two domains. *C*, the electrostatic surface map of C67S HisR shows a negatively charged active site. The *left panel* shows the complete electrostatic surface map of C67S HisR, indicating that the active site pocket displays an overall positive charge. The pocket can be identified in the middle of the structure *via* presence of the sulfate ion (*sticks*) situated between the two catalytic residues. The *middle panel* shows a partially transparent electrostatic surface map indicating the position of the active site residues, which are situated in the center of the structure at the ends of helices 2 (residue 67) and 4 (residue 209), and on either side of the sulfate ion. The *right panel* shows a close up view of the active site with its electrostatic surface map. ATCC, American Type Culture Collection; HisR, histidine racemase; PDB, protein data bank.

Structures of C67S and C209S HisR were very similar, with a backbone r.m.s.d. of 0.493 Å by aligning α-carbons of 260 residues (Fig. 4*A*), and each adopt a DapF-like fold. The DapF-like fold is an overall α+β structure, in which the monomer unit is composed of two structurally equivalent N-terminal and C-terminal domains (40, 41). The active site, consisting of two catalytic cysteine residues, can be found in the cleft between the N-terminal and C-terminal domains, each at the head of two central α-helices. The monomer unit is overall approximately pseudosymmetric and typically has low sequence identity between the N- and C-terminal domains. This fold has been characteristic of DAP and 4-hydroxyproline epimerases, proline racemase, and the only other currently characterized cofactor-independent histidine racemase: CntK from *S. aureus* (21, 42, 43). All of these features were found in the structure of HisR (Fig. 4*A*), with the N-terminal domain consisting of residues 1 to 121 and 246 to 265, while the C-terminal domain consists of residues 122 to 245 (Fig. 4*B*).

Each domain of HisR contains two sets of antiparallel β-sheets and two α-helices, much like CntK (Fig. S4). Catalytic residues 67 and 209 reside at the N-terminal ends of central α-helices 2 and 4, allowing for stabilization of the negative charge formed on cysteine thiolates by helix dipoles during the reaction (21). The substrate binding pocket of HisR has an overall negatively charged surface, and does not contain any basic residues (Fig. 4*C*). In each structure, a sulfate (mutant C67S) or a phosphate (mutant C209S) is found in the active site directly between catalytic residues. This is similarly found in the structure of CntK mutant C72S (PDB 6JIW), which contains a sulfate ion in the same position (22).

In DapF from *Corynebacterium glutamicum* (and other DAP epimerases), a major conformational change occurs upon substrate binding to bring the two domains together, close the binding pocket around the substrate, and exclude water from the active site (21, 44). The position of the loops above the active site spanning from residues 42 to 49 (N-terminal domain) and 197 to 206 (C-terminal domain) in HisR more closely match the conformation of the open *C. glutamicum* structure (Fig. S5*C*) than the substrate-bound, closed form (Fig. S5*D*). The HisR crystal structures obtained here closely match the AlphaFold2 structural prediction for this protein sequence (Fig. S5*E*), indicating that AlphaFold2 (https://alphafold.ebi.ac.uk/) models this enzyme in its open, substrate-unbound conformation (45).

**Figure 5:**
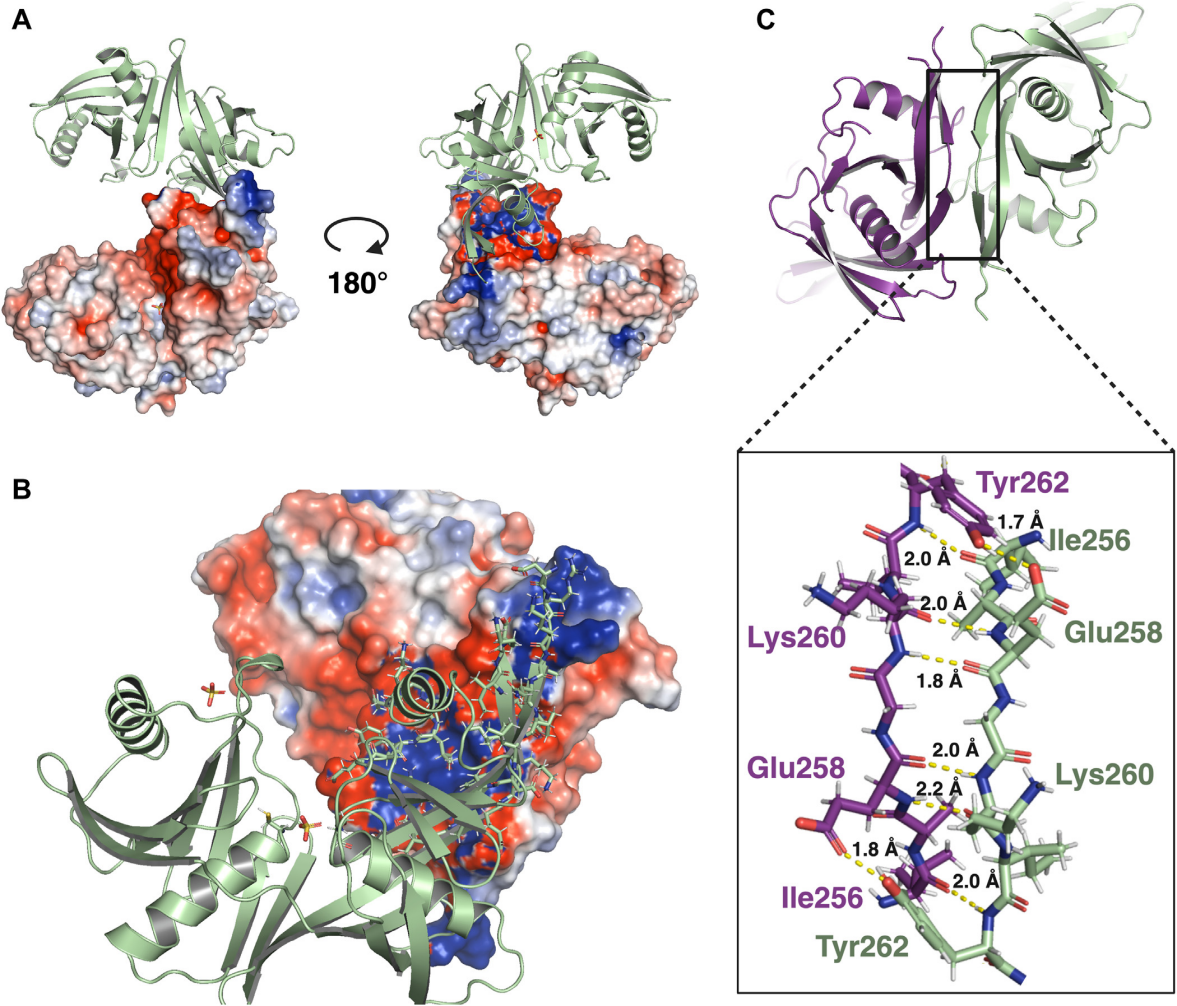
**The dimerization interface of HisR is composed of a symmetric, interchain β-sheet between residues 256 to 262.***A*, 3D view of the HisR dimer (C67S, PDB 9CR1), with electrostatic surface map shown. *B*, *front view* of HisR homodimer, with residues involved in dimerization shown as *sticks* contacting the electrostatic surface map of the second monomer. *C*, 3D view of dimerization surface with residues involved in close contacts between monomers shown. Regular β-sheet H-bonding patterns are found between the labeled residues, with H-bonding distances indicated. A single intermolecular side chain H-bond is also found between Tyr262 alcohol and Glu258 carboxylate in both monomers. HisR, histidine racemase; PDB, Protein Data Bank.

CntK and other enzymes with a DapF-like fold are typically functional as homodimers, and HisR appears to maintain this feature (Fig. 5) (22, 44, 46). The dimerization surface matches that seen in other enzymes of this class and is largely composed of interactions between C-terminal β-strands (β15, residues 256–262) to form an extended antiparallel β-sheet between the two monomer units, containing six interchain H-bonds. The surface is symmetric, and mirrors the dimerization surface observed in CntK. Beyond the regular H-bonding pattern formed between β-strands, there is a single side chain to side chain intermolecular H-bond formed between the phenol of Tyr262 and the carboxylate of Glu258.

### Prediction of interactions formed during histidine binding

**Figure 6:**
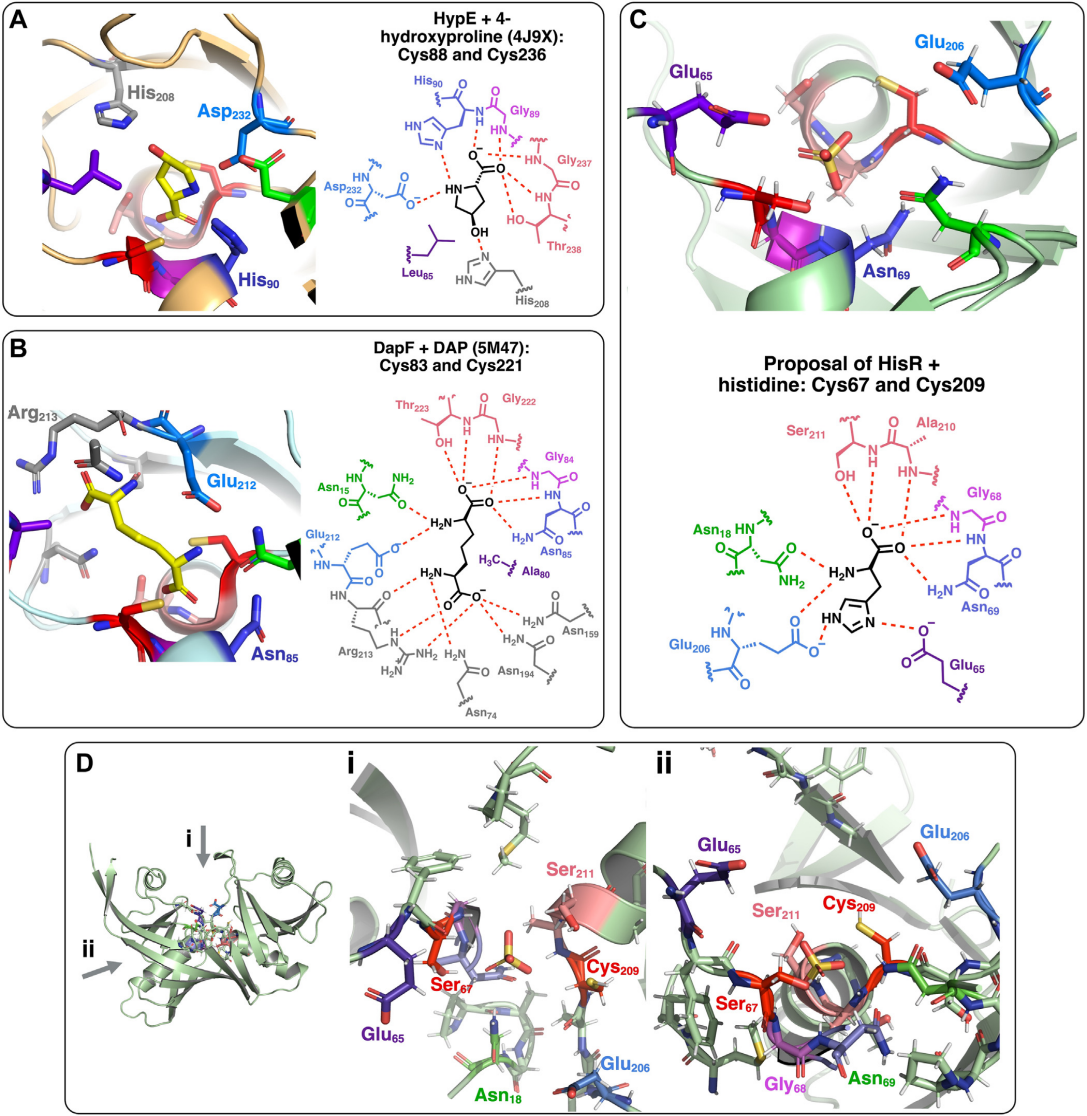
**Proposal of residues involved in HisR binding of histidine based on structural and sequence alignment with other cofactor-independent epimerases bound to substrates.***A*, binding of 4-hydroxyproline to HypE from *Pseudomonas protegens*. The *left panel* displays a crystal structure of the active site (PDS), and the *right panel* shows a color-coordinated illustration of the residues within H-bonding distance of the substrate. The residue colors correspond to the sequence and structural alignments of HypE, DapF, and HisR in Figures S6 and S7. Catalytic cysteine residues 88 and 236 are shown in *red*. *B*, binding of DAP to DapF from *Corynebacterium glutamicum* ATCC 13032. The *left panel* displays a crystal structure of the active site (PDB 5M47), and the *right panel* shows a color-coordinated illustration of the residues within H-bonding distance of the substrate. The residue colors correspond to the sequence and structural alignments of HypE, DapF, and HisR in Figures S6 and S7. Catalytic cysteine residues 83 and 221 are shown in *red*. *C*, proposal of binding of histidine to HisR. The *top panel* displays the crystal structure of the active site (PDB 9CR1), and the *bottom panel* shows a color-coordinated illustration of the residues proposed to be involved with substrate binding, based on structural and sequence alignment with HypE and DapF. The residue colors correspond to the sequence and structural alignments of HypE, DapF, and HisR in Figures S6 and S7. Catalytic cysteine 209 and mutated residue 67 (serine) are shown in *red*. *D*, proposal of HisR residues involved in binding of histidine, showing the active site from above and the side. The residue colors correspond to the sequence and structural alignments of HypE, DapF, and HisR in Figures S6 and S7. Catalytic cysteine 209 and mutated residue 67 (serine) are shown in *red*. ATCC, American Type Culture Collection; DAP, diaminopimelic acid; DapF, diaminopimelic acid epimerase; HisR, histidine racemase; HypE, hydroxyproline epimerase; PDB, Protein Data Bank.

In their report of the crystal structure of CntK, Luo *et al.* used ligand docking software to model the binding of histidine and CntK (22). However, as the structures of CntK and HisR show these enzymes in open, substrate-unbound forms, it is unclear whether the conformational change upon substrate binding will significantly affect a binding model generated based on the open form of the enzyme. Here, rather than using modeling software, we analyzed structures of DapF and hydroxyproline epimerase (HypE) in substrate-bound conformations to identify residues that may be involved in HisR binding of histidine. DapF and HypE were chosen because within cofactor-independent racemases and epimerases, DapF, HypE, and CntK/HisR are similar structurally, whereas glutamate and aspartate racemases comprise another structural group. We also specifically chose crystal structures of these enzymes (DapF and HypE) with substrates bound in the active site, and therefore forming the closed, substrate-bound enzyme conformations. First, we mapped residues within H-bonding distance of DAP and 4-hydroxyproline in crystal structures of DapF (*C. glutamicum*, PDB 5M47) and HypE (*Pseudomonas* sp., PDB 4J9X), respectively (Fig. 6, *A* and *B*). The positions of these residues were compared using a sequence alignment (Fig. S6) and structural alignment (Fig. S7). Residues potentially involved in HisR binding of histidine were then selected based on positional similarity to DapF and HypE residues involved in substrate binding (Figs. 6*C* and S7).

The positions of backbone atoms in DAP and 4-hydroxyproline substrates were highly conserved (Fig. S7*C*), and interactions with their respective epimerase enzymes were very similar. This led us to hypothesize that HisR may interact with the α-amino group of histidine through H-bonds with Asn18 and Glu206, while the α-carboxylate may form H-bonds with Gly68, Asn69, Ala210, and Ser211. While the side chains of 4-hydroxyproline and DAP are different, the position of several residues in proximity to side chain atoms are similar among these enzymes (Fig. S7*A*). In particular, Leu85 (HypE) and Ala80 (DapF) are within close proximity to substrate hydrophobic regions, and therefore the analogous residue Glu65 in HisR may form a H-bond with the δ-amino group of the imidazole side chain. The ε-amino group of histidine may form a H-bond with the γ-carboxylate of Glu206 in HisR, which occupies a similar location in the active site to Asp232 (HypE) and Glu212 (DapF), which each form H-bonds with their respective substrates.

### Comparison of HisR to CntK from *S. aureus*

**Figure 7:**
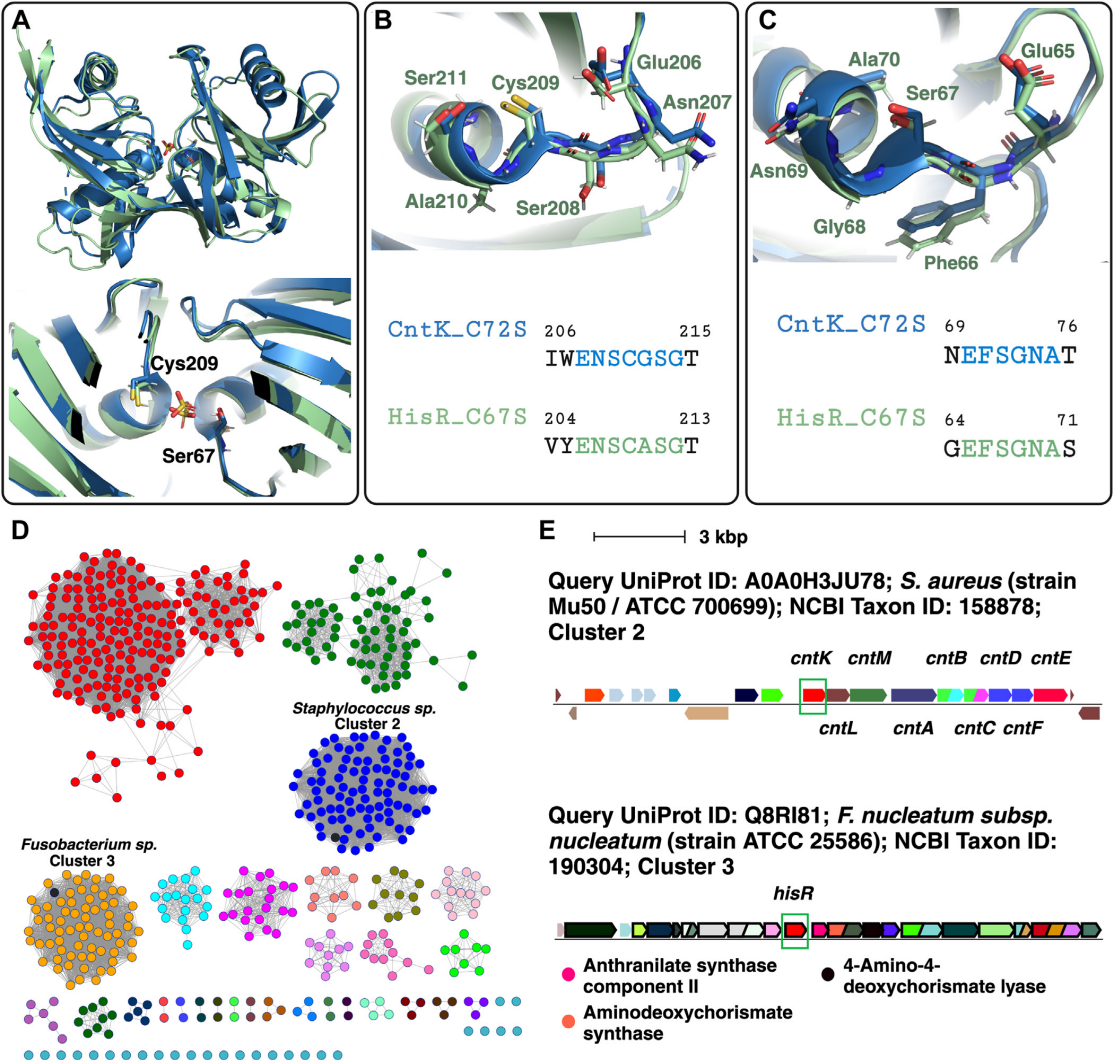
**Comparison of HisR from *Fusobacterium nucleatum* ATCC 25586 with CntK from *Staphylococcus aureus*, currently the only other reported cofactor-independent histidine racemase.***A*, overlay of crystal structures from *F. nucleatum* ATCC 25586 HisR C67S mutant (PDB 9CR1, *green*) with *S. aureus* Mu50 CntK C72S mutant (PDB 6JIW, *blue*). The overall r.m.s.d. (α-C) of the two structures is 1.412 Å. Both structures contain a sulfate ion situated in the active site pocket situated between the two catalytic residues. The residues labeled in *green* are based on the HisR protein sequence. *B*, active site residues flanking catalytic cysteine 209 in HisR (*green*), overlaid with active site residues of CntK flanking cysteine 211 (*blue*). The residues labeled in *green* are based on the HisR protein sequence. *C*, active site residues flanking mutated serine 67 in HisR (*green*), overlaid with active site residues of CntK flanking serine 72 (*blue*). The residues labeled in *green* are based on the HisR protein sequence. *D*, sequence similarity network of HisR from *F. nucleatum* ATCC 25586. The network (617 nodes and 18,197 edges; representative nodes shown based on 100% identity) was constructed with a threshold sequence identity for drawing edges of ∼40% (alignment score 55), minimum length 250 residues, and maximum length 308 residues. Cluster 2 contains primarily proteins from *Staphylococcus sp.*, while cluster 3 contains primarily proteins from *Fusobacterium sp.* CntK from *S. aureus* Mu50 and HisR from *F. nucleatum* ATCC 25586 are indicated as *black* ellipses in respective clusters. *E*, genome neighborhood diagrams for *Staphylococcus aureus* Mu50 CntK and *F. nucleatum* ATCC 25586 HisR. Histidine racemase genes are indicated with *green boxes*. The complete list of genes upstream and downstream of both histidine racemase genes are described in Table 3. Here, only the *S. aureus cnt* biosynthetic gene cluster is labeled, while for *F. nucleatum* only the genes that appear in the genome neighborhood network (Table 4) with >80% cooccurrence with HisR in cluster 3 are indicated. ATCC, American Type Culture Collection; HisR, histidine racemase; PDB, Protein Data Bank.

Currently, the only other cofactor-independent histidine racemase characterized is CntK from *S. aureus* (22, 23). This enzyme racemizes L-to D-histidine for use in biosynthesis of the broad-spectrum metallophore staphylopine. Overlay of the structures of CntK and HisR showed that while these two enzymes have relatively low sequence identity (28%), their structures are overall very similar, especially within the active site (Fig. 7, *A*–*C*). To determine whether other predicted cofactor-independent histidine racemases exist in nature, a sequence similarity network (SSN) was generated using HisR as a search model (47, 48). A SSN represents protein sequences as circles called nodes, and pairwise identity between protein sequences as lines called edges. The user can specify a minimum threshold sequence identity to connect nodes with edges, and as the threshold is increased edges are removed and the nodes will separate into distinct groups called clusters (Fig. 4, *B*–*F*). Within each cluster the sequences are similar and may indicate isofunctional proteins. Assigning the functionality of proteins in different clusters is more difficult and relies on experimental evidence, as different clusters may represent isofunctional proteins with lower sequence identity, or may represent proteins with entirely different functions.

The SSN generated based on HisR used an initial UniProt BLAST query e-value of 5, with a maximum of 1000 sequences retrieved. The network shown in Figure 7*D* contains 617 nodes and 18,197 edges and was generated with a threshold sequence identity of ∼40% (alignment score 55), with representative nodes shown containing 100% identity. With this threshold, CntK and HisR are not clustered together (28% identity). Since these are the only two currently experimentally validated PLP-independent histidine racemases, the function of proteins in clusters other than 2 and 3 are difficult to interpret, especially because cofactor-independent epimerases and racemases often have low sequence identity within and between enzyme types. However, it is likely that enzymes within cluster 2 (from *Staphylococcus* sp.) and cluster 3 (from *Fusobacteria* sp.) are also histidine racemases, based on high sequence identity with CntK and HisR, respectively.

We were interested to see whether *hisR* was located in a biosynthetic gene cluster potentially involved in biosynthesis of staphylopine, or another similar opine metallophore. Staphylopine and other metallophores are important molecules that can increase bacterial virulence by enabling the organism to chelate ions essential for survival away from the host (24, 25). However, upon examination of the genome of *F. nucleatum* ATCC 25586, we found that in addition to *hisR* being located in an entirely different genome context than *cntK* (Table 3 and Fig. 7*E*), homologues of the other genes involved in staphylopine biosynthesis (*cntL* and *cntM*) were absent in the genome (10). This suggests that these two enzymes play different roles in *F. nucleatum* and *S. aureus.* We then generated a genome neighborhood network (±10 ORFs, minimum cooccurrence 20%) for each gene in order to examine whether genomic contexts were conserved among *Staphylococcus* sp. and *Fusobacterium* sp. in clusters 2 and 3, respectively (49). This genome neighborhood network showed that homologues of genes involved in staphylopine biosynthesis have high cooccurrence rate with homologues of *cntK* in cluster 2, while the genes surrounding *hisR* homologues are less highly conserved (Table 4). Only three genes had cooccurrence ratios above 80% in cluster 3, and whether the predicted encoded enzymes (class IV aminotransferase/aminodeoxychorismate lyase, class I glutamine amidotransferase/aminodeoxychorismate synthase component II, anthranilate/aminodeoxychorismate synthase component I) are related to *hisR* is unclear.

**Table 3:** Annotation of genes from upstream to downstream (±10) of *hisR* and *cntK* in *F. nucleatum* ATCC 25586 and *Staphylococcus aureus* Mu50

*Staphylococcus aureus* ATCC 700699; cluster 2; CntK	*Fusobacterium nucleatum* ATCC 25586; cluster 3; HisR
DUF1413 domain-containing protein (A0A0H3JT89)	V-type sodium ATP synthase subunit G (Q8RI71)
Uncharacterized oxidoreductase SAV2478 (Q99RF5)	V-type ATP synthase subunit I (Q8RI72)
DUF1433 domain-containing protein (A0A0H3JTL9)	V-type sodium ATP synthase subunit K (Q8RI73)
DUF1433 domain-containing protein (A0A0H3K113)	V-type ATP synthase subunit E (Q8RI74)
DUF1433 domain-containing protein (A0A0H3JU84)	V-type sodium ATP synthase subunit C (Q8RI75)
Carboxymuconolactone decarboxylase-like domain-containing protein (A0A0H3JXB4)	V-type ATP synthase subunit F (Q8RI76)
Similar to aminobenzoyl-glutamate transport protein (A0A0H3JT84)	V-type sodium ATP synthase subunit A (Q8RI77)
Similar to glucose 1-dehydrogenase (A0A0H3JTL6)	V-type ATP synthase beta chain (Q8RI79)
Metallo-beta-lactamase domain-containing protein (A0A0H3K107)	V-type ATP synthase subunit D (Q8RI80)
**Histidine racemase CntK (A0A0H3JU78)**	**Histidine racemase HisR (annotated as diaminopimelate epimerase DapF;****Q8RI81****)**
D-histidine 2-aminobutanoyltransferase CntL (A0A0H3JXA8)	Anthranilate synthase component II (Q8RI82)
Staphylopine synthase CntM (A0A0H3JT80)	Aminodeoxychorismate synthase (Q8R6F3)
Metal-staphylopine-binding protein CntA (A0A0H3JTL0)	4-Amino-4-deoxychorismate lyase (Q8R6F4)
Metal-staphylopine import system permease protein CntB (A0A0H3K104)	Pyrrolidone-carboxylate peptidase (Q8RI83)
Metal-staphylopine import system permease protein CntC (A0A0H3JU73)	Chloride channel protein (Q8RI84)
Metal-staphylopine import system ATP-binding protein CntD (A0A0H3JXA3)	Multidrug export protein MepA (Q8RI85)
Metal-staphylopine import system ATP-binding protein CntF (A0A0H3JT74)	Potassium uptake protein KtrB (Q8RI86)
Staphylopine export protein CntE (A0A0H3JTK0)	Potassium uptake protein KtrA (Q8RI87)
Transposase (A0A0H3K100)	tRNA uridine 5-carboxymethylaminomethyl modification enzyme MnmG 2 (Q8RI88)
CPBP family intramembrane metalloprotease (A0A0H3JU68)	Ribosomal RNA small subunit methyltransferase G (Q8RI89)

UniProt IDs for each protein are indicated in brackets. Histidine racemase genes are in bold.

**Table 4:** Genome neighborhood networks for Clusters 2 (*Staphylococcus sp.*) and 3 (*Fusobacterium sp.*)

SSN cluster number	Name	Pfam description	Cooccurance	Cooccurance ratio
2	adh_short	Short chain dehydrogenase	0.37	32/86
**2**	**Staph_opine_DH**	**Staphylopine dehydrogenase**	**0.98**	**85/86**
2	DUF1433	Protein of unknown function (DUF1433)	0.38	33/86
**2**	**MFS_1**	**Major facilitator superfamily**	**0.97**	**84/86**
2	adh_short_C2	Enoyl-(acyl carrier protein) reductase	0.34	30/86
2	Glu_synthase	Conserved region in glutamate synthase	0.32	28/86
2	CMD	Carboxymuconolactone decarboxylase family	0.38	33/86
**2**	**BPD_transp_1-OppC_N**	**Binding-protein-dependent transport system inner membrane component-N-terminal TM domain of oligopeptide transport permease C**	**0.95**	**82/86**
**2**	**BPD_transp_1-BPD_transp_1_N**	**Binding-protein-dependent transport system inner membrane component-binding-prot-dependent transport system membrane comp, N-term**	**0.95**	**82/86**
**2**	**SBP_bac_5**	**Bacterial extracellular solute-binding proteins, family 5 middle**	**0.97**	**84/86**
2	ABG_transport	AbgT putative transporter family	0.39	34/86
**2**	**ABC_tran**	**ABC transporter**	**0.97**	**84/86**
3	GidB	rRNA small subunit methyltransferase G	0.47	33/70
**3**	**Chorismate_bind-Anth_synt_I_N**	**Chorismate binding enzyme-Anthranilate synthase component I, N terminal region**	**0.85**	**60/70**
3	Voltage_CLC-TrkA_C	Voltage gated chloride channel-TrkA-C domain	0.62	44/70
3	V_ATPase_I	V-type ATPase 116 kDa subunit family	0.57	40/70
3	TrkA_C-TrkA_N	TrkA-C domain-TrkA-N domain	0.58	41/70
3	TMP-TENI	Thiamine monophosphate synthase	0.22	16/70
3	Peptidase_C15	Pyroglutamyl peptidase	0.61	43/70
3	PDDEXK_9-AAA-ATPase_like	PD-(D/E)XK nuclease superfamily-Predicted AAA-ATPase	0.21	15/70
3	MatE	MatE	0.61	43/70
**3**	**GATase**	**Glutamine amidotransferase class-I**	**0.85**	**60/70**
3	GIDA-GIDA_C-GIDA_C_1st	Glucose inhibited division protein A-tRNA modifying enzyme MnmG/GidA C-terminal helical bundle-tRNA modifying enzyme MnmG/GidA C-terminal helical domain	0.57	40/70
3	TrkH	Cation transport protein	0.61	43/70
**3**	**Aminotran_4**	**Amino-transferase class IV**	**0.81**	**57/70**
3	ATP-synt_D	ATP synthase subunit D	0.61	43/70
3	ATP-synt_C	ATP synthase subunit C	0.58	41/70
3	ATP-synt_ab-ATP-synt_ab_N-ATP-synt_ab_Xtn	ATP synthase alpha/beta family, nucleotide-binding domain-ATP synthase alpha/beta family, beta-barrel domain-ATPsynthase alpha/beta subunit N-term extension	0.6	42/70
3	ATP-synt_ab-ATP-synt_ab_N	ATP synthase alpha/beta family, nucleotide-binding domain-ATP synthase alpha/beta family, beta-barrel domain	0.61	43/70
3	ATP-synt_F	ATP synthase (F/14-kDa) subunit	0.61	43/70
3	vATP-synt_E	ATP synthase (E/31 kDa) subunit	0.61	43/70
3	vATP-synt_AC39	ATP synthase (C/AC39) subunit	0.61	43/70

Bolded are genes with neighborhood cooccurrence with the query gene (*cntK* for cluster 2 or *hisR* for cluster 3) above 80%.

### Growth experiments of *F. nucleatum* ATCC 23726

Finally, we were interested to see whether a knockout version of the *hisR* gene in *F. nucleatum* has any effect on growth or survival of the organism. While the HisR enzyme characterized in the above work is from strain ATCC 25586, *F. nucleatum* ATCC 23726 is easier to genetically manipulate with a wide variety of tools that are available. A thiamphenicol resistance gene in a Tn*5* transposition as the selection marker was used to create a *ΔhisR* strain of *F. nucleatum* ATCC 23726 (Fig. S10). To justify using this *ΔhisR* strain of ATCC 23726 for our growth studies, we first needed to confirm that the *hisR* gene in this organism actually encodes a histidine racemase. The ATCC 23726 HisR protein is 92% identical to that of HisR from 25586 (Fig. S8). After cloning and expression of the *hisR* gene from ATCC 23726 in *E. coli*, and isolation of the enzyme, we confirmed that it does indeed function as a histidine racemase with very similar kinetic parameters to HisR from strain ATCC 25586 (Fig. S9 and Table S2). Additionally, the genome neighborhood surrounding ATCC 23726 *hisR* is highly similar to that of ATCC 25586 *hisR*, suggesting these enzymes maintain the same function in both organisms.

**Figure 8:**
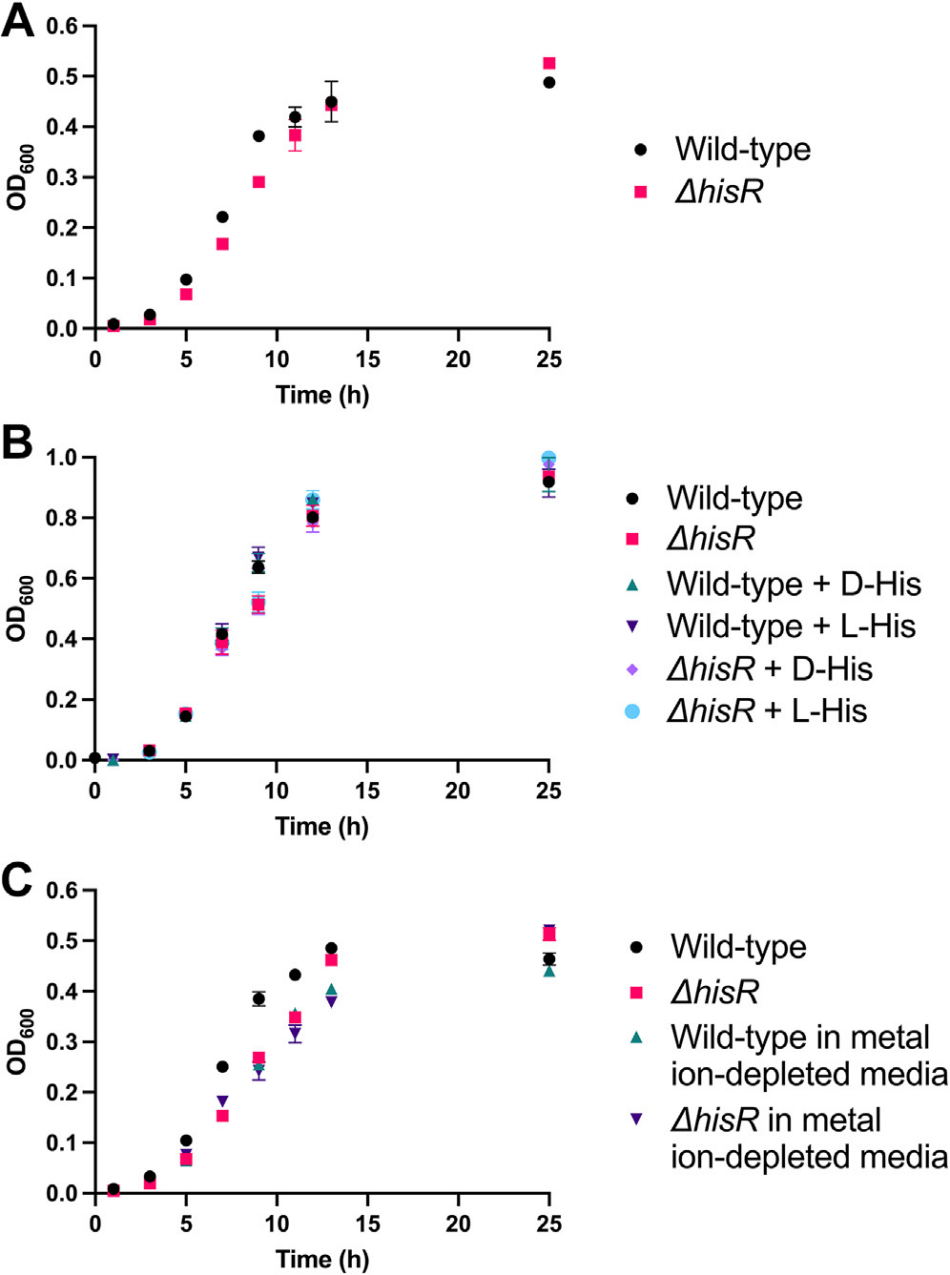
**Growth curves of WT and *ΔhisR* strains of *Fusobacterium nucleatum* ATCC 23726.***A*, comparison of growth of *F. nucleatum* ATCC 23726 WT and *ΔhisR*. *B*, comparison of growth of *F. nucleatum* ATCC 23726 WT and *ΔhisR* in the presence of 1.5 mM D- or L-histidine. *C*, comparison of growth of *F. nucleatum* ATCC 23726 WT and *ΔhisR* in regular or metal ion-depleted media. ATCC, American Type Culture Collection; HisR, histidine racemase.

We could observe a visual difference in WT and *ΔhisR* cultures. While overnight cultures of the WT organism were suspended throughout the liquid growth media, the *ΔhisR* cultures were visually clumpy and not suspended evenly, instead growing at the bottom and sides of the culture tube. We were also able to observe a small but consistently reproducible difference in growth of WT and *ΔhisR* strains of *F. nucleatum* ATCC 23726. The *ΔhisR* organism lagged behind the WT during late exponential phase, which was observed by monitoring *A*_600_ of cell cultures over time (Fig. 8*A*). We then tried to see whether addition of D-histidine to the culture media could recover the growth rate of *ΔhisR* to that of the WT, but this did not lead to any measurable difference (Fig. 8*B*). Finally, we grew both strains on metal-ion depleted media (pretreated with NTA) in order to determine if the abundance of metal ions had any effect on the growth of the two strains. Here, we found that depletion of metal ions from the media resulted in the growth curve of the WT organism approaching that of the *ΔhisR* strain (Fig. 8*C*). This preliminary result requires future investigation, but may suggest that like CntK in *S. aureus*, *F. nucleatum* HisR might play a role in metal ion acquisition and growth of the organism.

## Discussion

In this work, we characterized only the second so far identified cofactor-independent histidine racemase, HisR. This enzyme had remarkable specificity for histidine, and substrates bearing even minor modifications of the backbone or imidazole ring such δ-O oxazole, ε-S thiazole, and pyrrole structures were not tolerated by HisR, suggesting that H-bonding requirements within the imidazole ring are critical for substrate recognition. It is therefore surprising that the enzyme can also epimerize lanthionine, albeit at a greatly reduced rate (Fig. 1 and Table 1). While the best substrate for an enzyme is not necessarily the physiologically relevant one, it is interesting to note that knockout of *hisR* is not a lethal mutation. On the other hand, knockout of lanthionine synthase (*cysK1*, FN1220) in *F. nucleatum* ATCC 23726 causes cell death, and survival can be recovered by addition of lanthionine to the growth media (50). This suggests that HisR is not necessary for lanthionine biosynthesis, but it is possible that it still plays some role in this pathway.

Despite being an overall well-studied class of enzymes, cofactor-independent racemases and epimerases are limited so far to just a few substrates: DAP, aspartate, glutamate, 2,4-diaminobutyric acid, proline, 4-hydroxyproline, *O-*ureidoserine, isoleucine, and histidine, with relatively few crystal structures solved (21, 42). This lack of structural information is because members of this enzyme class are typically difficult to crystallize, probably due to high mobility and large conformational changes that occur upon substrate binding. One “trick” that has been used to solve this problem is mutation of one of the catalytic cysteines to a serine residue (39). This mutation hinders catalysis, but probably helps to keep the enzyme in its open conformation, thereby aiding crystallization. However, an enzyme crystallized in this way will not provide much information about interactions between the substrate and enzyme. It is also useful to note that AlphaFold2 seems to model these enzymes, including HisR, in the open, substrate-unbound conformation with overall high confidence (pLDDT >90 for >90% of all residues). In this study, we found that structural analysis using Phyre2 (http://www.sbg.bio.ic.ac.uk/∼phyre2/html/page.cgi?id=index) and FoldSeek (https://search.foldseek.com/search), rather than sequence analysis (pBLAST), was a more accurate method of function prediction.

The histidine binding model for CntK from *S. aureus* proposed by Luo *et al.* is similar to that proposed here for HisR, except in the identity of residues potentially forming interactions with histidine’s side chain (22). In the CntK model, the authors proposed Glu208 (analogous to Glu206 in HisR) and Glu46 (analogous to Glu49 in HisR) as key residues forming H-bonds with the imidazole ring. In our proposal for HisR, we also identified Glu206 as a potentially key residue, but rather than Glu49, we propose Glu65 as the second H-bonding residue with the histidine imidazole. This prediction was based on sequence and structural alignments to substrate-bound HypE and DapF enzymes, where analogous residues Leu85 and Ala80 are closely positioned to their substrate side chains, respectively. In both cases, the side chains of 4-hydroxyproline and DAP extend outward to position side chain atoms directly between these hydrophobic residues and Asp232 or Glu212 (analogous to Glu206 in HisR), respectively. Luo *et al.* did conduct a thermal shift assay on a E46A mutant of CntK and observed an impact on substrate binding, but this could also be explained by perturbation of overall active site architecture.

There are currently only two other histidine racemases characterized in nature: the PLP-independent histidine racemase CntK from *S.* aureus (23), and the recently identified PLP-dependent histidine racemase from lactic acid bacterium *Leuconostoc mesenteroides* subsp. sake NBRC 102480 (51). The biological role of the PLP-dependent enzyme is unknown, but the role of CntK in producing D-histidine for staphylopine biosynthesis is well studied (24, 25). Interestingly, knockout of *cntK* in *S. aureus* significantly reduces virulence of this organism in mice (52), and so we were interested to examine whether HisR is involved in metallophore production in *Fusobacteria*. However, *F. nucleatum* lacks the other genes required for staphylopine biosynthesis, and *hisR* is surrounded by an entirely different set of genes than *cntK*, suggesting that HisR and CntK provide D-histidine for different metabolic products useful for optimal growth.

Ramezani *et al.* observed in 1999 that *F. nucleatum* can deplete both D- and L-histidine from nutrient media, but this ability was not ubiquitous for all D-amino acids (53). With this in mind, we expected that addition of D-histidine to bacterial media may recover the growth rate of a *ΔhisR F. nucleatum* strain to WT levels, but this was not observed. The purpose of HisR in *F. nucleatum* remains therefore unclear, but it is interesting to note that in other *Fusobacteria* species, the three genes that cooccur (>80%) with HisR homologues are all predicted to be involved in folate biosynthesis. Whether HisR and these other enzymes are involved together in a biological process is unknown, but it may be possible that D-histidine has an overlooked role in *F. nucleatum*, setting the stage for future studies into the physiological role of HisR in this organism.

## Experimental procedures

### Molecular biology

DNA coding sequences for *F. nucleatum* ATCC 25586 (GenBank ID: AAL93847.1; UniProt ID: Q8RI81) and *F. nucleatum* ATCC 23726 (GenBank ID: EFG94488.1; UniProt ID: D5RFM4) histidine racemases with C-terminal hexahistidine tags were codon optimized for expression in *E. coli* using Integrated DNA Technologies Codon Optimization Tool. Genes were synthesized by GenScript and inserted into pET-24b(+) cloning vectors using a 5′ *Nde*I and a 3′ *Hind*III restriction site. Plasmids were then transformed into *E. coli* BL21(DE3) (New England BioLabs) according to the manufacturer instructions, and cultures were stored as 20% glycerol stocks at −80 °C.

HisR active site mutants were generated using the Q5 Site-Directed Mutagenesis Kit (New England BioLabs). DNA primers were supplied by Integrated DNA Technologies. To generate the C67S HisR mutant from *F. nucleatum* ATCC 25586, forward primer 5′-GGGGAATTTTcTGGCAATGCTTC-3′ and reverse primer 5′-TCCCATCATTTGCAGATG-3′ were used, while the C209S HisR mutant was generated with forward primer 5′-GAGAATAGTTcTGCGTCAGGTAC-3′ and reverse primer 5′-ATACACTCCAGACCCAAC-3′. Generated plasmids were transformed into *E. coli* DH5α (New England BioLabs), and then plasmid DNA was purified and sequenced. Plasmids with the appropriate mutations were then transformed into *E. coli* BL21(DE3) for protein production, and cultures were stored as 20% glycerol stocks at −80 °C.

### Protein expression and enzyme purification

Frozen glycerol stocks of *E. coli* BL21(DE3) cells transformed with the appropriate pET-24b(+) vector containing the *hisR* gene or its mutated derivative were inoculated into 50 ml of sterile Difco LB Broth, Miller media with kanamycin (50 μg/ml) as selective pressure. The cells were grown overnight at 37 °C with shaking at 240 rpm. The next day, 20 ml of the overnight culture was added to 500 ml of sterile LB media with kanamycin added, and cells were grown to an absorbance (*A*_600_) of ∼0.8 at 37 °C. Protein expression was then induced at 37 °C by addition of isopropyl-β-D-1-thiogalactopyranoside (Chem-Impex International) to a final concentration of 0.5 mM, and the flasks were vigorously shaken for an additional 4 h. Cells were then harvested by centrifugation (5000*g*, 10 min, 4 °C) and the pellets were stored at −80 °C.

Frozen cell pellets were resuspended (30 ml buffer/500 ml culture media) evenly in ice cold lysis buffer (50 mM NaH_2_PO_4_, 300 mM NaCl, and 5 mM imidazole, pH 8.0) and lysed by sonication while kept on ice. DNase I (Thermo Fisher Scientific, 1 U) was added and the lysate kept on ice for 30 min with occasional inversion. The cellular debris was removed by centrifugation (20,000*g*, 30 min, 4 °C), and then Ni-NTA resin (McLab) was added to the clarified supernatant, and the mixture was gently shaken for 1 h at 4 °C. The mixture was then loaded onto a fritted column, and the flow through collected at 4 °C. The resin was washed with 25 ml of lysis buffer containing 20 mM imidazole, and then the fusion protein was eluted in 5 ml fractions by sequential addition of elution buffer (50 mM NaH_2_PO_4_, 300 mM NaCl, pH 8) containing 40, 60, 80, 100, 200, and then 500 mM imidazole. Eluted fractions were analyzed by SDS-PAGE and the samples containing the protein of interest were pooled together and then loaded on a Sephadex G-15 size exclusion column (50 mM NaH_2_PO_4_, 150 mM NaCl, pH 8 for enzyme assays or 20 mM Tris, 100 mM NaCl, 2 mM DTT, pH 8 for crystallography). Elution of the protein of interest was monitored by absorbance at 280 nm, and then fractions were pooled and concentrated using an Amicon Ultra Centrifugal Filter, 10 kDa molecular weight cut-off (5000*g*, 4 °C). Protein concentration was measured by absorbance at 280 nm using a Nanodrop spectrophotometer. Aliquots of the enzyme solutions were stored at −80 °C.

### Substrate screening with D_2_O NMR assay

To screen potential substrates for HisR enzymes, solutions of amino acids (1–5 mM) were dissolved in D_2_O phosphate buffer (600 μl, 20 mM NaH_2_PO_4_, 0.1 mM 2-mercaptoethanol, pD 7.3) at room temperature. To initiate the reaction, enzyme diluted in D_2_O phosphate buffer (∼2 μg) was added. The ^1^H-NMR spectrum was recorded at given times from addition of the enzyme (typically at ∼15 min and again at 48 h). Spectra were analyzed for disappearance of the α-H compared to that of a negative control without added enzyme, prepared from the same original amino acid solution. NMR spectra were collected on a Varian 600 MHz NMR spectrometer, processed with vnmrj, and then spectra lines were darkened and text enlarged using Inkscape 1.3.2 (https://inkscape.org/).

### Circular dichroism

All reactions were monitored using a 0.2 mm quartz cuvette at 25 °C using an OLIS globalworks CD spectrophotometer. The maximum CD signal was obtained for histidine and lanthionine at 212 nm, and all reactions were monitored at this wavelength. The average integrated CD signal (in mdeg) was recorded every five seconds. Reactions were conducted in phosphate buffer (20 mM NaH_2_PO_4_, 100 mM NaCl, pH 7.5). All experiments were performed in duplicate, except for experiments to determine kinetic parameters, which were performed in triplicate.

The data were plotted as the average of the values recorded in two or three experiments, and for kinetic experiments the error displayed is the average of the mean. For all experiments, the reaction was initiated by addition of HisR, and then directly placed in the spectrophotometer. The data were processed in Excel and GraphPad Prism (https://www.graphpad.com/features). For overshoot experiments, buffers were prepared in deuterated water. For kinetic experiments, a calibration curve was prepared for both D- and L-histidine by averaging the CD signal obtained over a five-minute period for a concentration range of 2 to 40 mM. The data were plotted in a calibration curve, and the concentrations (mM) were interpolated from the curve. The initial linear range was used to perform Michaelis–Menten analysis in GraphPad Prism.

### X-ray crystallography

Each HisR mutant was concentrated to 39 mg/ml before screening crystallization suites. Crystallization conditions were screened by mixing 0.5 μl protein with 0.5 μl reservoir using Phoenix ARI crystallization Robot (Art Robbins Instruments), and crystals were grown at 18 °C in a sitting-drop plate. After three days, crystals of HisR C67S appeared in the condition containing 0.1 M Hepes pH 7.4, 2% PEG 400, 2 M ammonium sulfate, while crystals of HisR C209S appeared in the condition of 0.1 M phosphate-citrate pH 4.2, 40% PEG 300. The crystals were flash frozen in liquid nitrogen after being passed through 20% glycerol as a cryoprotectant.

The data were collected at Stanford Synchrotron Radiation Light Source, at beamline 12-1. The data were processed using the program XDS (https://xds.mr.mpg.de/) (54). The molecular placement was done by the program CCP4 phaser MR (55, 56) using a structure prediction by AlphaFold (45) as a searching model (Fig. S5*E*). The structures were refined using Phenix (https://phenix-online.org/) (57) and modified manually in Coot (https://www2.mrc-lmb.cam.ac.uk/personal/pemsley/coot/) (58). Data statistics and PDB IDs are summarized in Table 2.

### Bioinformatic analysis

Protein sequence alignments were performed using UniProt Align, and structural alignments were performed using RSCB PDB Pairwise Structure Alignment Tool (59). To generate sequence alignments including secondary structure elements, protein sequences were aligned using T-Coffee (https://tcoffee.crg.eu/) (60), and then secondary structure elements from crystal structures PDB 9CR1 (C67S HisR) and 6JIW (CntK C72S) were overlaid with this sequence alignment using ESPript 3.0 (61). Results were overlaid using Inkscape 1.3.2.

SSNs and genome neighborhood networks were generated using the EFI-EST tool (49, 62). The SSN for HisR (Fig. 7*D*) was produced using an initial UniProt BLAST query e-value of 5, with a maximum of 1000 sequences retrieved. The resulting network contained 617 nodes and 18,197 edges, using a threshold sequence identity of ∼40% (alignment score 55), minimum length 250 residues, and maximum length 308 residues. The representative nodes shown contain 100% identity. The genome neighborhood network was generated from the SSN using a neighborhood size of ten and cooccurrence ratio of 20%.

### Generation of ΔhisR strain of *F. nucleatum* ATCC 23726

The *ΔhisR* strain of *F. nucleatum* ATCC 23726 was generated as previously described using Tn*5* transposon mutagenesis (63). The *hisR* gene (FN1732) is located in an orphan operon, and the single-primer one-step PCR mapping of the Tn*5* insertion site in this transposon mutant is at the 281 nucleotide position of the gene, which has a total length of 792 nucleotide. The transposon insertion in this gene was verified using PCR, with forward primer 5′-ATGGATGGAAAAGTGCAAGTTTTAGAT-3′ and reverse primer 5′-TCATATGTACACCTTTCCTTCTGCT-3′. Using these primers, the WT organism produces a PCR product of 798 bp, while the mutant produces a product of 2053 bp.

### *F. nucleatum* ATCC 23726 growth experiments

Base media for *F. nucleatum* growth experiments consisted of 3% BD BBL Trypticase Soy Broth (Soybean-Casein Digest Medium) and 1% Bacto Peptone. Subsequently, 8 ml of media was aliquoted into culture tubes and then autoclaved. A fresh solution of 1% cysteine was prepared daily, adjusted to pH 7, filter sterilized, and then 400 μl was added to each sterilized culture tube to a final concentration of 0.05%. After addition of cysteine, culture tubes were immediately placed in an AS-150 anaerobic chamber (Anaerobe Systems) in an atmosphere of nitrogen (90%), carbon dioxide (5%), and hydrogen (5%). *F. nucleatum* ATCC 23726 WT and *ΔhisR* cultures were inoculated from 20% glycerol stocks into the prepared media and then grown for 15 h at 37 °C in an anaerobic environment to reach stationary phase. Overnight cultures were then inoculated into fresh media at a concentration of 1% (v/v) and grown for varying time lengths at 37 °C under anaerobic conditions. *A*_600_ was monitored by removing samples at various time points, and then measuring absorbance at 600 nm using either 1 ml of sample in a 1.5 ml disposable polystyrene cuvette and a Genesys 10S Vis Spectrophotometer (Thermo Fisher Scientific), or 200 μl of sample in a 96-well plate with a SpectraMax i3x plate reader (Molecular Devices). The data were processed in GraphPad Prism. All growth experiments were completed in duplicate, subtracted against a blank, and the error displayed is the average of the mean. For experiments monitoring the effect of added histidine, L- or D-histidine (30 mM) + cysteine (1%) solutions were prepared fresh daily, adjusted to pH 7, filter sterilized, and then 400 μl was added to culture tubes to final concentrations of 1.5 mM and 0.05%, respectively.

To prepare metal ion-depleted media, Ni-NTA agarose (25 ml, McLab) was washed with 200 ml of 100 mM EDTA (pH 7) and then with 250 ml of distilled water to remove Ni^2+^. The beads were then washed with 20% ethanol, and then added to 200 ml of 3% trypticase soy broth and 1% peptone media (filter sterilized). The solution was shaken overnight at 4 °C, and then filter sterilized to remove the agarose beads, adjusted to pH 7, aliquoted into 8 ml culture tubes, and autoclaved. Overnight cultures for metal ion-depleted media experiments were grown in this media.

## Data availability

All data are contained within the manuscript and its supporting information files.

## Supporting information

This article contains supporting information (64, 65, 66).

## Conflict of interest

The authors declare that they have no conflicts of interest with the contents of this article.
