# Supplementary material for: Discovery, characterization, and structure of a cofactor-independent histidine racemase from the oral pathogen Fusobacterium nucleatum
Source: J Biol Chem. 2024 Oct 17;300(11):107896. doi: 10.1016/j.jbc.2024.107896 (PMC11602996; doi:10.1016/j.jbc.2024.107896)
Supplement: Supporting Information [file mmc1.docx]

***Supporting Information for “Discovery, characterization, and structure of a cofactor-independent histidine racemase from the oral pathogen Fusobacterium nucleatum”***

Tess Lamer^1^, Pu Chen^2,3^, Marie J. Venter^1^, Marco J. van Belkum^1^, Anjalee Wijewardane^1^, Chenggang Wu^4^, M. Joanne Lemieux^2,3^, John C. Vederas^1^*

^1^ University of Alberta, Department of Chemistry, Edmonton, AB, T6G 2G2, Canada

^2^ University of Alberta, Department of Biochemistry, Edmonton, AB, T6G 2H7 Canada

^3^ Li Ka Shing Institute of Virology, University of Alberta, Edmonton, AB, T6G 2E1, Canada

^4^ University of Texas McGovern Medical School, Department of Microbiology and Molecular Genetics, Houston, TX, 77030, USA

Table S1 2

Figure S1 3

Figure S2 4

Figure S3 5

Figure S4 6

Figure S5 7

Figure S6 8

Figure S7 9

Figure S8 10

Figure S9 11

Table S2 12

Figure S10 12

Supporting Information Methods 13

Protein and DNA Sequences 13

Construction of Tn*5* Mutant in *F. nucleatum* ATCC 23726 15

Synthesis of Oxazole Analogue of Histidine 17

Supporting Information References 18

Table S1**.** *F. nucleatum* ATCC 25586 protein function predictions for FN1732 based on amino acid sequence or AlphaFold2 structural model.

|  | **pBlast** | **Phyre2** | **FoldSeek (PDB100)** |
| --- | --- | --- | --- |
| **Top protein function prediction based on amino acid sequence** | Diaminopimelate epimerase | Histidine racemase (*Staphylococcus aureus* CntK; PDB 6JIS) | Histidine racemase (*Staphylococcus aureus* CntK; PDB 6JIS) |

**
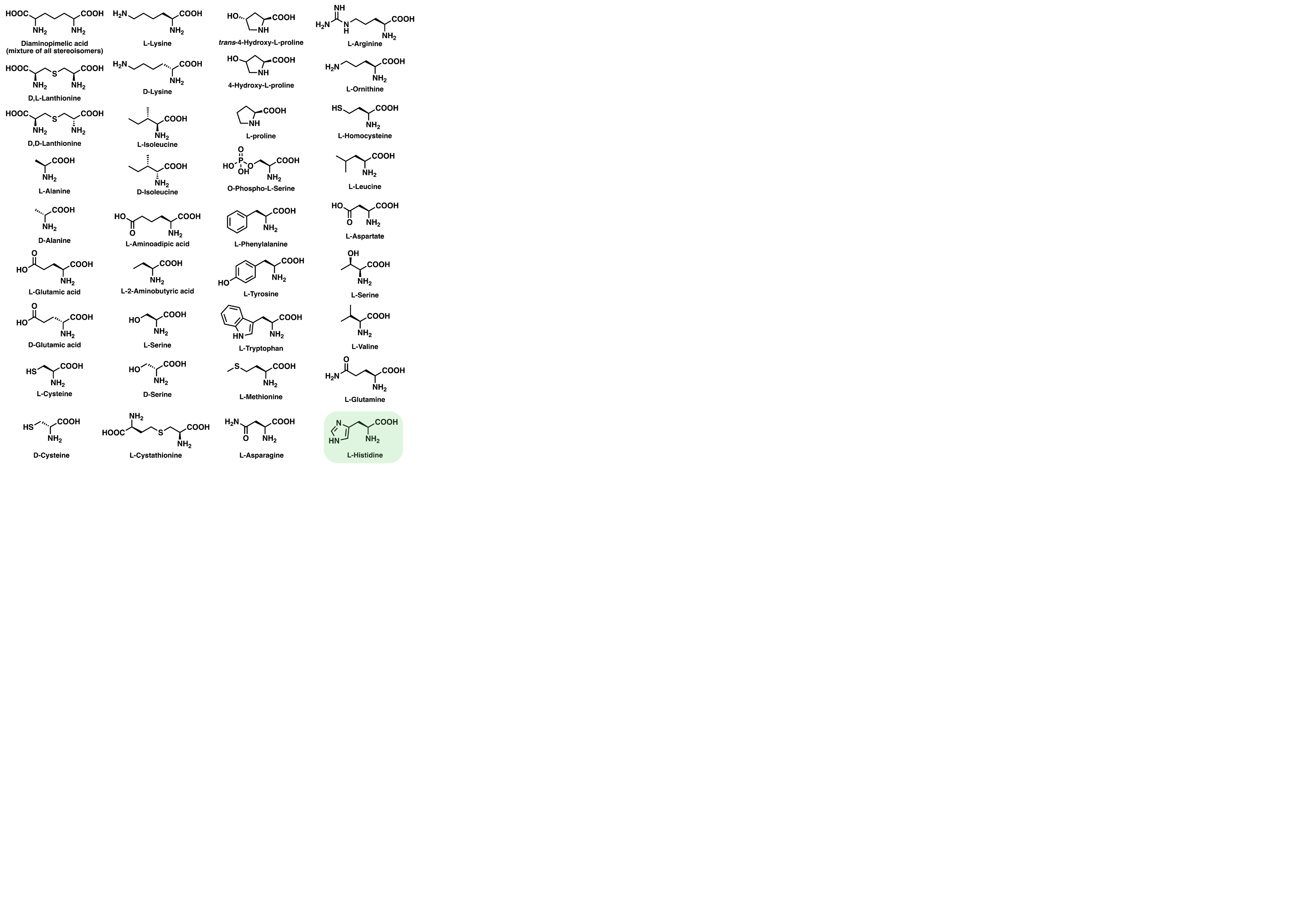
**

Figure S1**. Amino acids tested as substrates with *F. nucleatum* ATCC 25586 HisR in D_2_O ^1^H-NMR assay.** α-H signal disappearance was not observed after 48 h for any of the shown compounds, except histidine, when HisR was added to the solution. The α-H signal of histidine completely disappeared via replacement with deuterium in less than 15 minutes in the same assay.


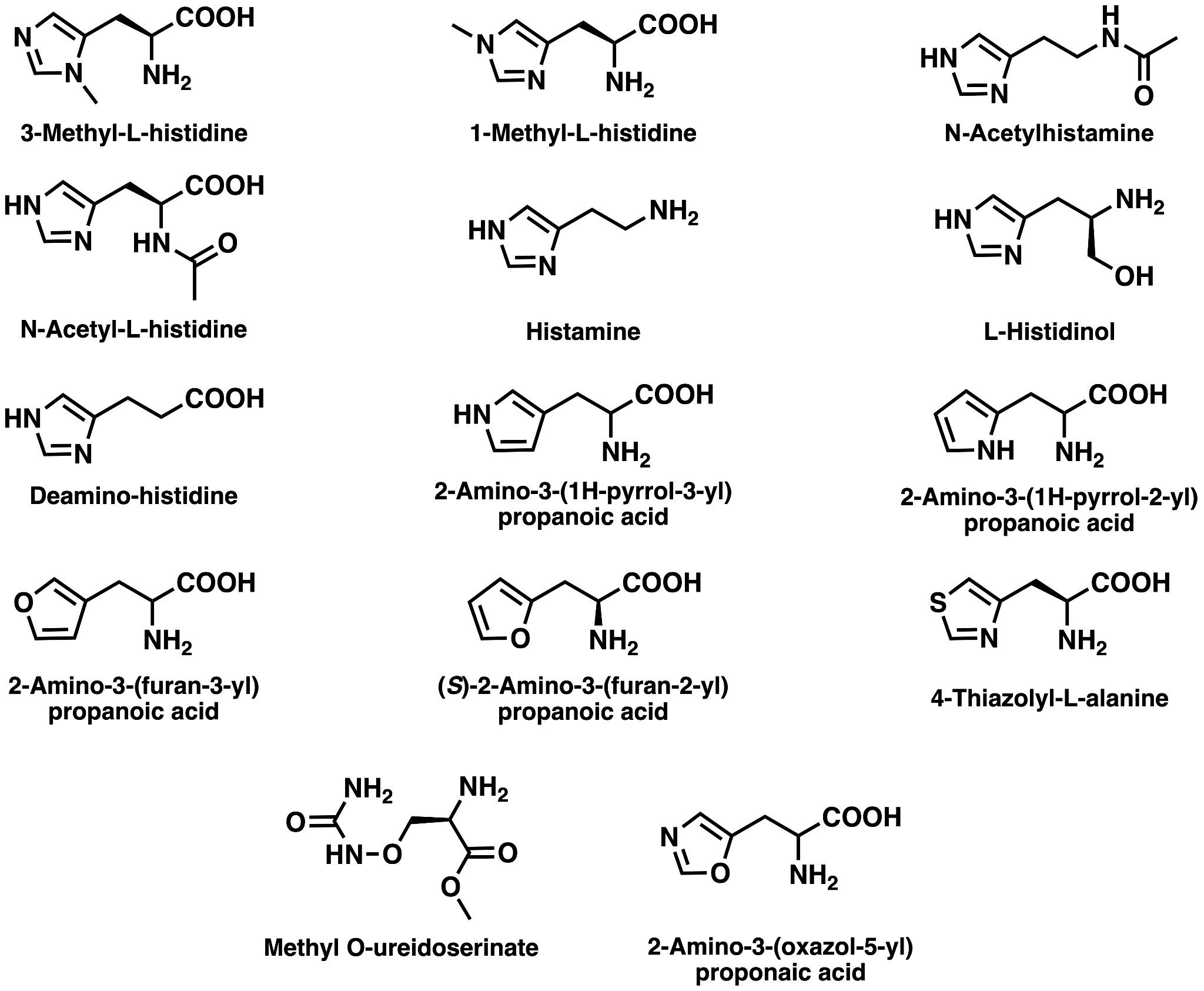


Figure S2**. Histidine analogues tested as substrates with *F. nucleatum* ATCC 25586 HisR in D_2_O ^1^H-NMR assay.** α-H signal disappearance was not observed after 48 h for any of the shown compounds when HisR was added to the solution, while the α-H signal of histidine completely disappeared via replacement with deuterium in less than 15 minutes in the same assay.

**
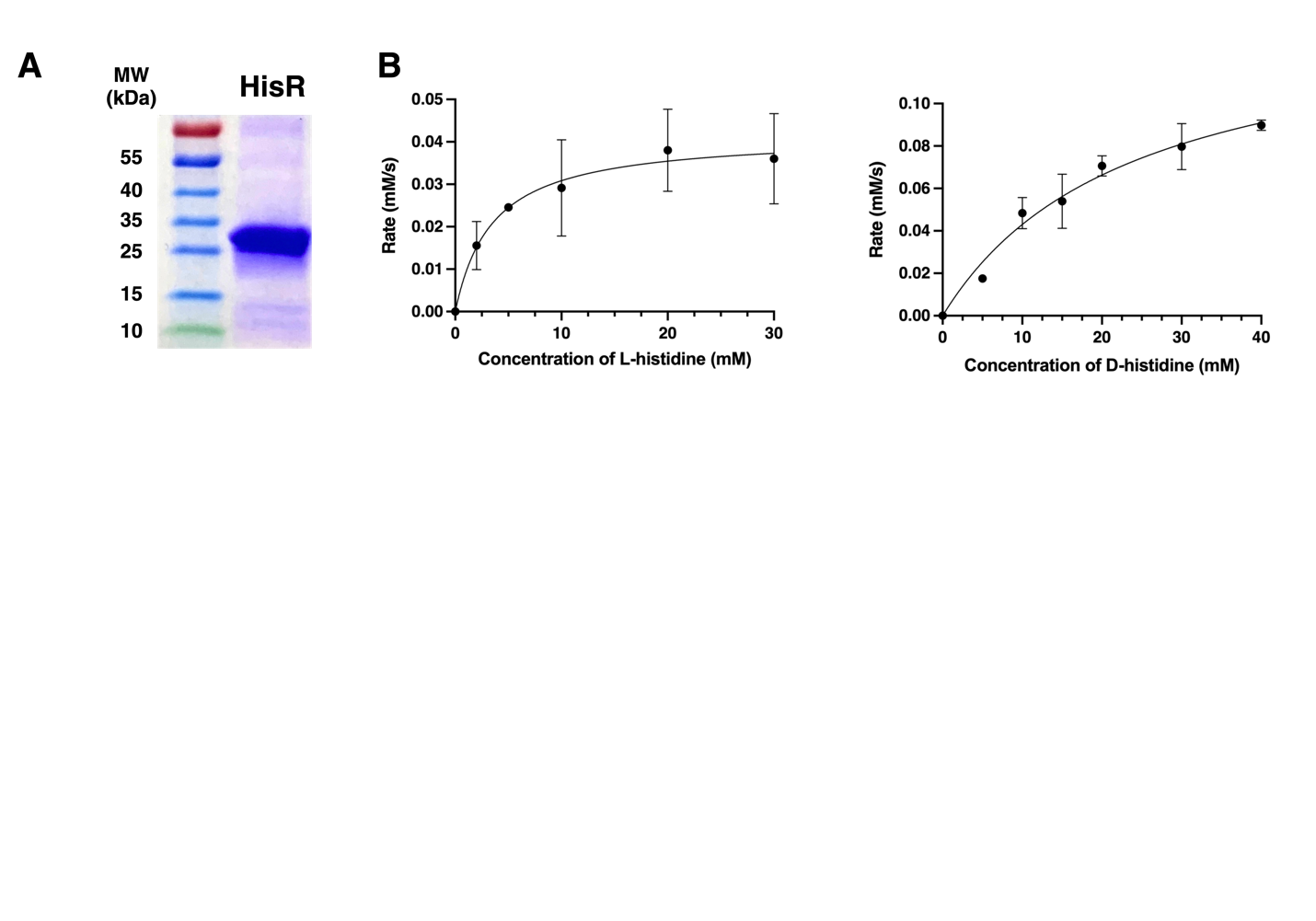
**

Figure S3**. Purification and activity of HisR from *F. nucleatum* ATCC 25586. A)** SDS-PAGE of purified HisR from *F. nucleatum* ATCC 25586 after heterologous expression in *E. coli* BL21(DE3). **B)** Michaelis-Menten plots for *F. nucleatum* strain 25586 histidine racemase. Kinetic experiments were performed using circular dichroism (mdeg over time) with wavelength monitoring at 212 nm. Each CD signal was obtained over an integration time of 5 seconds, and mdeg values were converted to concentration using a standard curve. Experiments were performed in triplicate with varying concentrations of D- or L-histidine, and the results of the three trials were averaged to obtain kinetic constants.


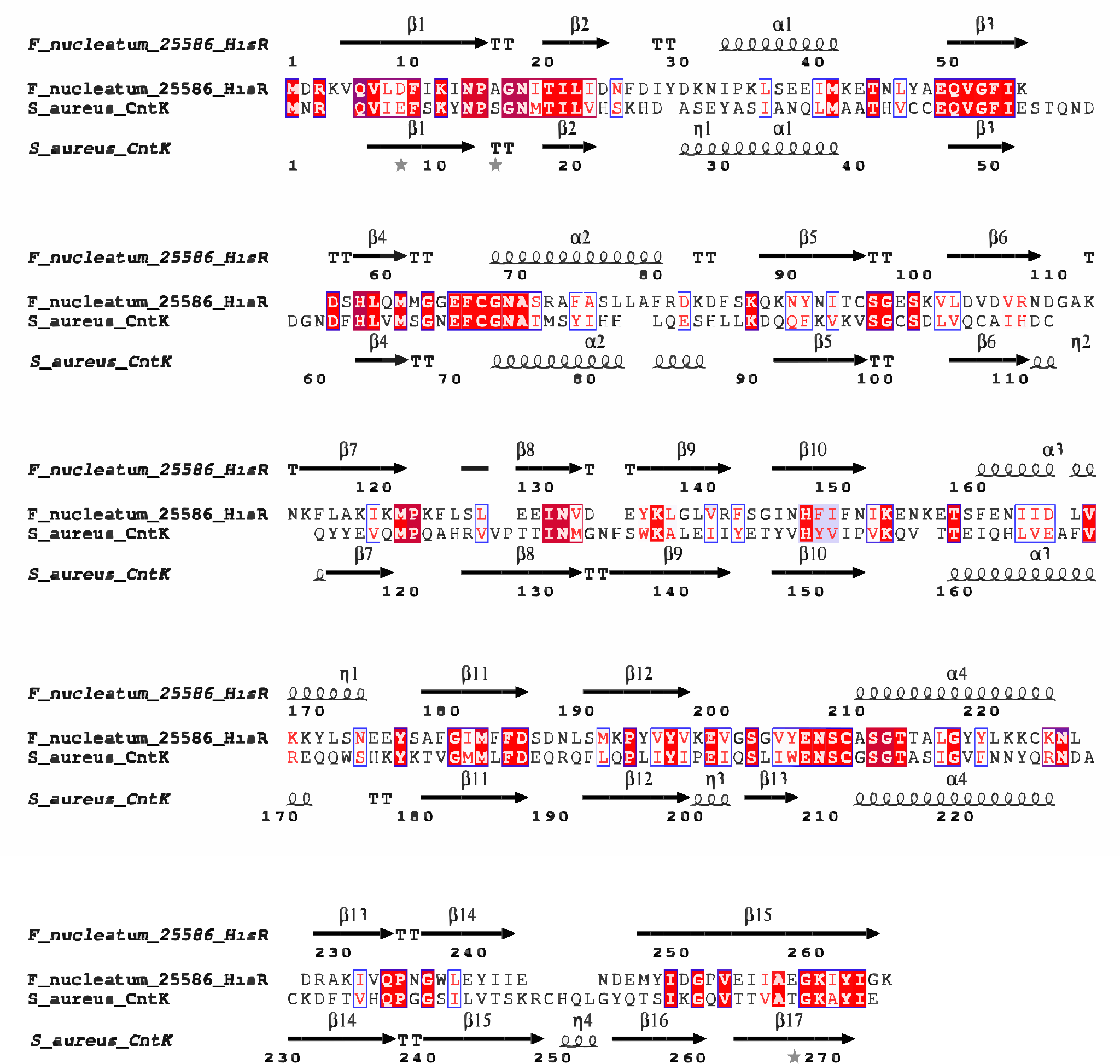


Figure S4**. Amino acid sequence alignments of histidine racemases from *F. nucleatum* ATCC 25586 and *S. aureus* Mu50.** Secondary structure elements mapped to each protein are shown, based on crystal structures of mutants C67S (*F. nucleatum*, PDB 9CR1) and C72S (*S. aureus*, PDB 6JIW), respectively.

**
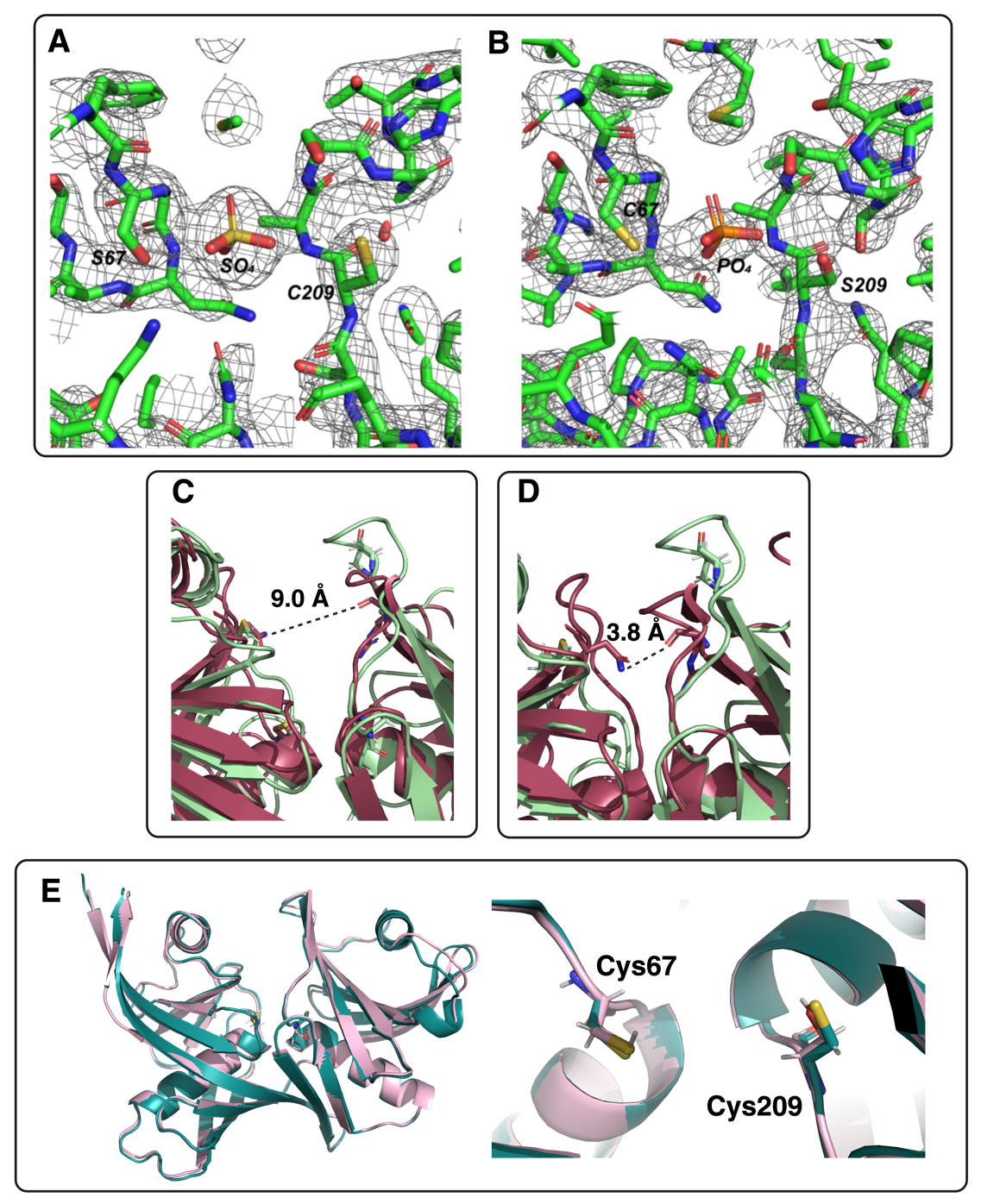
**

Figure S5**. Crystal structures and AlphaFold structure prediction of HisR are in the open, substrate-unbound form.** *A*, Composed omit map of catalytic site of HisR C67S generated by phenix and visualized by pymol at a contour level of 2σ. *B,* (A) Composed omit map of catalytic site of HisR C209S generated by phenix and visualized by pymol at a contour level of 2σ. *C,* Overlay of *Corynebacterium glutamicum* DAP epimerase in open, substrate-unbound form (PDB: 5H2Y, raspberry) with HisR C67S (PDB 9CR1, green). *D*, Overlay of *Corynebacterium glutamicum* DAP epimerase in closed, substrate bound form (PDB: 5M47, raspberry) with HisR C67S (PDB 9CR1, green). *E*, Overlay of *F. nucleatum* ATCC 25585 C209S crystal structure (PDB 9CR6, pink) and AlphaFold2 structure prediction (turquoise) indicates that AlphaFold2 models this enzyme in open, substrate-unbound form. The overall r.m.s.d. (α-C) of the two structures is 0.504 Å.

**HisR_Staphylococcus_aureus_CntK_6JIS** ----------------------------------------MNRQVIEFSKYNPSG**N**MTIL 20

**HisR_Fusobacterium_nucleatum_25586** --------------------------------------MDRKVQVLDFIKINPAGNITIL 22

**HypE_Pseudomonas_protegens_4J9X** ------------------------------------------MKKITVIDSHTGGEPTRL 18

**DapF_Corynebacterium_glutamicum_5M47** -----------------------------------------MNLTIPFAKGHATEND-FI 18

**HisR_Staphylococcus_aureus_CntK_6JIS**  VHSKHD-ASEYASIANQLMAATHVCC**EQ**-----------------------------VGF 50

**HisR_Fusobacterium_nucleatum_25586** IDNFDIYDKNIPKLSEEIMKETNLYA*EQ*-----------------------------VGF 53

**HypE_Pseudomonas_protegens_4J9X**  VIDG------FPDLGRGSMAERLQILEREHDQWRRACV-LEPRGSD*V*---------*LV*GA 62

**DapF_Corynebacterium_glutamicum_5M47**  IIPD------EDA---------RLDLTP--EMVVTLCDRRAGIGA*DG*ILRVVKAADVEGS 61

**HisR_Staphylococcus_aureus_CntK_6JIS** IESTQNDDGNDFHLVM----SGNEFC**GN**ATMSYIHHL--QESHLLKDQ--QFKVKVSGCS 102

**HisR_Fusobacterium_nucleatum_25586**  IK--------DSHLQM----MGGEFCGNASRAFASLLAFRDKDFSKQK--NYNITCSGES 99

**HypE_Pseudomonas_protegens_4J9X**  LLCQPQAGDACAGVIFFNNSGYLGMCGHGTIGLVRSLYHLGRIDQGV------HRIETPV 116

**DapF_Corynebacterium_glutamicum_5M47**  T-VDP--SL-WFMDYRNADGSLAEMCGNGVRLFAHWLYSRGLVDNTS------FDIGTRA 111

**HisR_Staphylococcus_aureus_CntK_6JIS**  DLVQCAIHDCQ--------YYEVQMPQAHRVVPTT-INMGNHSWKALEIIYETYVHYVIP 153

**HisR_Fusobacterium_nucleatum_25586** KVLDVDVRNDGAK---NKFLAKIKMPKFLSLE--E-IN--VDEYKLGLVRFSGINHFIFN 151

**HypE_Pseudomonas_protegens_4J9X**  GTVEATLHE-------DLSVSVRNVPAYRYRTQ-VMLQLPGHGKVHGDIAWGGNWFFLIS 168

**DapF_Corynebacterium_glutamicum_5M47** GVRHVDILQ----ADQHSAQVRVDMGI-----P----DVTGLST----C--------DIN 146

**HisR_Staphylococcus_aureus_CntK_6JIS**  VK----------------------QVTTEIQHLVEAFVREQQWSHKY-----KTVGMMLF 186

**HisR_Fusobacterium_nucleatum_25586**  IK----------------------ENKETSFENIIDLVKKYLSNEEY-----SAFGIMFF 184

**HypE_Pseudomonas_protegens_4J9X**  DH---GQRIALD---------NVEALTHYTRDVRQAL-EAAGITGAEGGVI---DHIELF 212

**DapF_Corynebacterium_glutamicum_5M47** GQVFAGLGVDMGNPHLACVVPGLSASALADMELRAP-----TFDQEFFPHGVNVEI---- 197

**HisR_Staphylococcus_aureus_CntK_6JIS** DEQRQFLQPLIYIPEIQSLIW-**E**NSC**GS**GTASIGVFNNYQRNDACKDFT--VHQPGGSIL 243

**HisR_Fusobacterium_nucleatum_25586**  DSDNLSMKPYVYVKEVGSGVY-ENSCASGTTALGYYLKKCKNLD--RAK--IVQPNGWLE 239

**HypE_Pseudomonas_protegens_4J9X** --ADDPQADSRNFVLCPGKAYDRSPCGTGTSAKLACLAADGKLAPGQAWRQASVIGSQFS 270

**DapF_Corynebacterium_glutamicum_5M47** --VTELEDDAVSMRVWERGVGETRSCGTGTVAAACAALADAGLGEGTVK--VCVPGGEVE 253

**HisR_Staphylococcus_aureus_CntK_6JIS** VTSKRCHQLGYQ--------TSIKGQVTTVATGKAYIE---------------------- 273

**HisR_Fusobacterium_nucleatum_25586** YIIE-----NDE--------MYIDGPVEIIAEGKIYIGK--------------------- 265

**HypE_Pseudomonas_protegens_4J9X**  AHYE---KVGE--------------QLIPILRGSAHISAEATLLLDDSDPFVWGIGS--- 310

**DapF_Corynebacterium_glutamicum_5M47** VQIFDDGS------T-------------------LT--GPSAIIALGEVQI--------- 277

Figure S6**. Multiple sequence alignment of histidine racemases CntK and HisR with 4-hydroxyproline epimerase (HypE) and DAP epimerase (DapF).** Residues highlighted in HypE and DapF are shown in respective substrate-bound, closed-form crystal structures to H-bond with the respective substrate. Corresponding residues are highlighted in the HisR sequence and proposed to be involved in substrate binding. Bolded residues in the CntK sequence were proposed to be involved with histidine binding by Luo *et al.* (22).


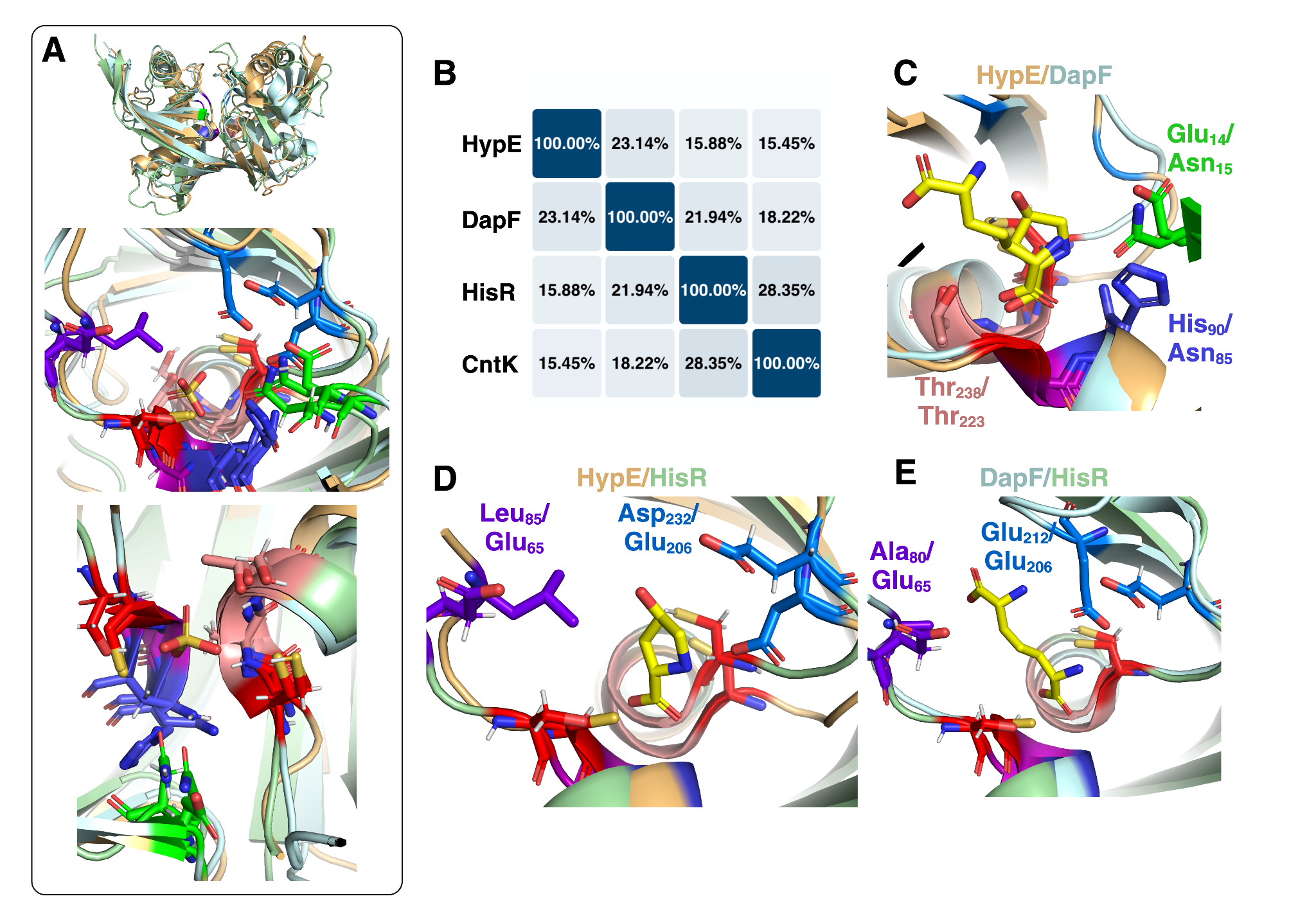


Figure S7**. Structural alignment of HypE, DapF, and HisR.** *A*, Structural alignment of HypE from *Pseudomonas protegens* (PDB 4J9X), DapF from *Corynebacterium glutamicum* (PDB 5M47), and HisR C67S (PDB 9CR1) from *F. nucleatum* ATCC 25586. The top panel displays the overall structural alignment, and the bottom two panels show that the residues predicted to be involved in HisR binding of histidine (Fig. S6 and Fig. 6) are generally in the same locations within the three structures. *B*, Percent identity matrix of HypE, DapF, HisR, and CntK. *C*, Structural alignment of HypE (orange) and DapF (cyan) with residues involved in interactions with backbone atoms of the substrates indicated. The position of α-amino and α-carboxylate in 4-hydroxyproline and DAP in these structures is almost identical, suggesting that binding interactions of backbone amino and carboxyl groups may be conserved amongst cofactor-independent epimerase or racemase enzymes. *D*, Structural alignment of HypE (orange) and HisR C67S (green), with HypE residues involved in interactions with the side chain of 4-hydroxyproline shown as sticks, along with catalytic cysteine residues shown in red. The aligned residues in HisR are also shown, and found to be in similar locations, suggesting these residues may be involved in interactions with the imidazole side chain of histidine. *E*, Structural alignment of DapF (cyan) and HisR C67S (green), with DapF residues involved in interactions with the distal end of DAP shown as sticks, along with catalytic cysteine residues shown in red. The aligned residues in HisR are also shown, and found to be in similar locations, suggesting these residues may be involved in interactions with the imidazole side chain of histidine.


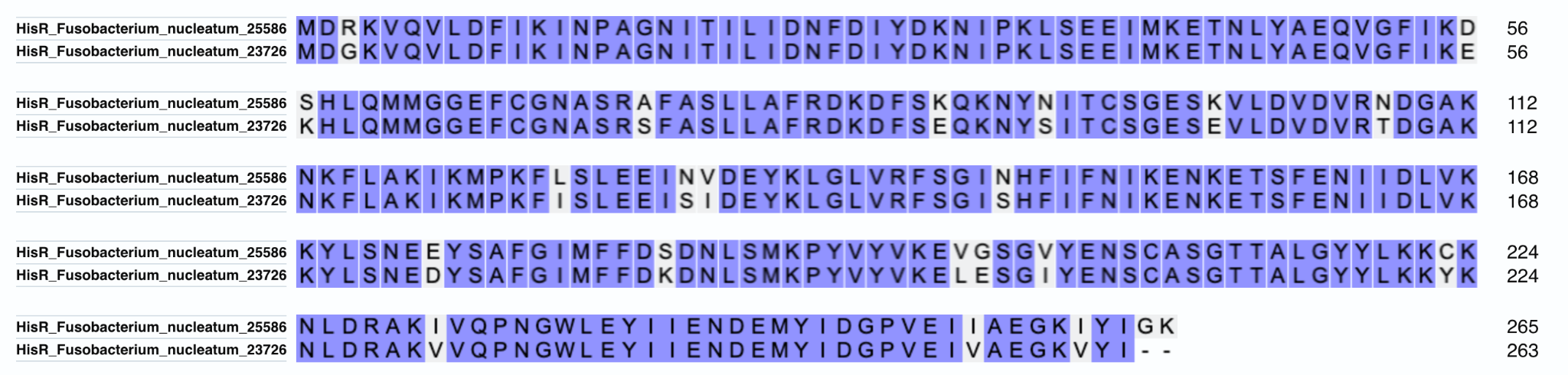


Figure S8**. Sequence alignment of *F. nucleatum* histidine racemase enzymes from ATCC 25586 (UniProt ID Q8RI81) and ATCC 23726 (UniProt ID D5RFM4), which share 92% sequence identity.**


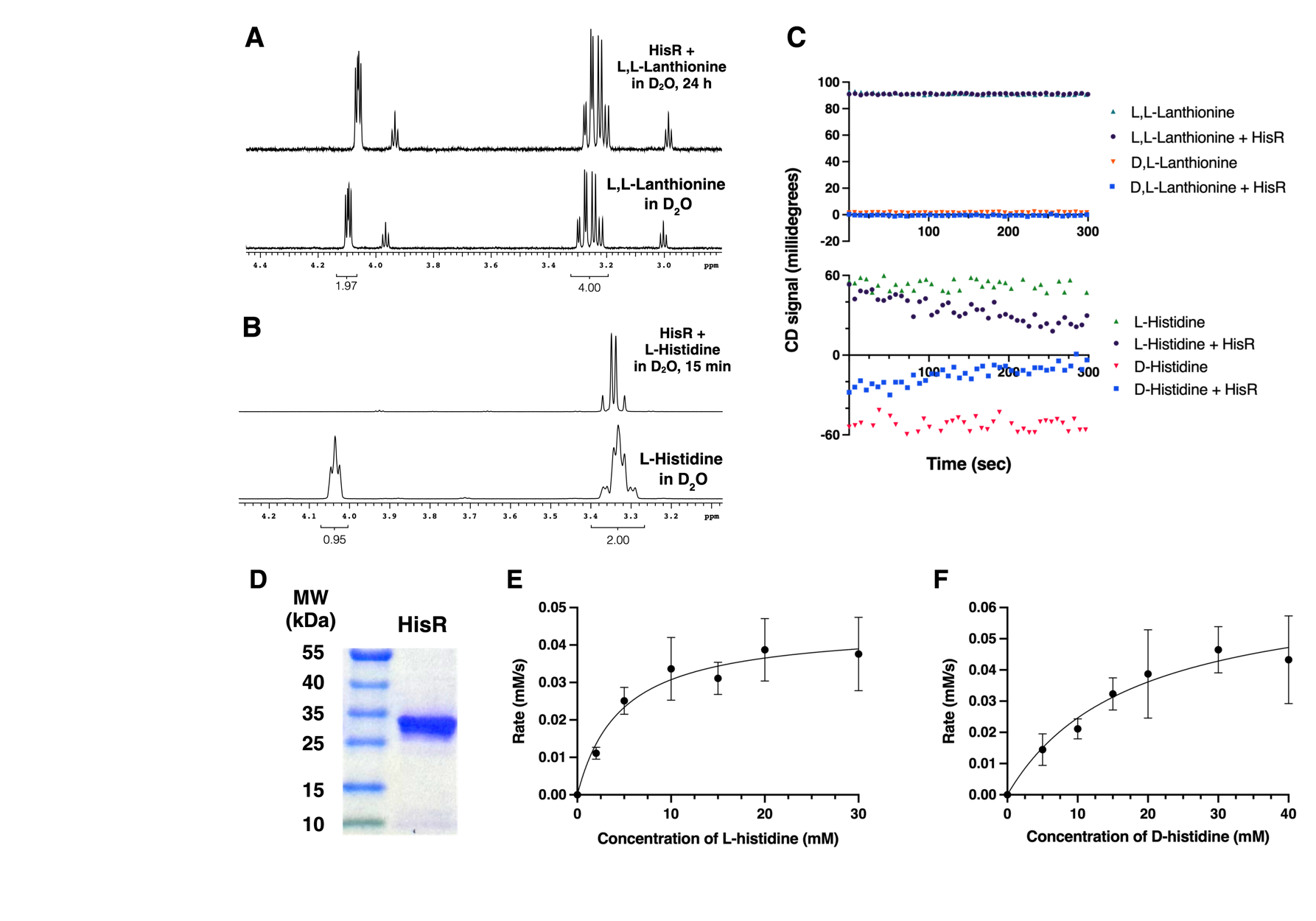


Figure S9**. Purification and activity of HisR from *F. nucleatum* ATCC 23726.** *A*, D_2_O ^1^H-NMR assay of *F. nucleatum* ATCC 23726 HisR with L,L-lanthionine. Solutions of 5 mM L,L-lanthionine in D_2_O buffer were prepared and analyzed with a 600 MHz NMR spectrometer. ~3 µg of HisR enzyme diluted in D_2_O buffer was added, and spectra were collected again after 24 h. The small triplets at 3.96 and 3.00 ppm are from 2-mercaptoethanol, which was added to experiments longer than 8 h in order to keep HisR reduced. *B*, ^1^H-D_2_O NMR assay of *F. nucleatum* ATCC 23726 HisR with L-histidine. Solutions of 5 mM L-histidine in D_2_O buffer were prepared and analyzed with a 600 MHz NMR spectrometer. ~3 µg of HisR enzyme diluted in D_2_O buffer was added, and spectra were collected again after 15 min. *C*, Circular dichroism monitoring of *F. nucleatum* ATCC 23726 HisR enzyme (5 µg) activity with 30 mM solutions of either lanthionine or histidine. The CD signal was recorded at 212 nm, and the average integrated CD signal in mdeg was recorded every five seconds. Experiments were performed in duplicate and CD signals displayed are the average between the two trials. *D*, SDS-PAGE of purified HisR from *F. nucleatum* ATCC 23726 after heterologous expression in *E. coli* BL21(DE3). *E*, Michaelis-Menten plot for *F. nucleatum* ATCC 23726 histidine racemase enzyme with L-histidine. Kinetic experiments were performed using circular dichroism (mdeg over time) with wavelength monitoring at 212 nm. Each CD signal was obtained over an integration time of 5 seconds, and mdeg values were converted to concentration using a standard curve. Experiments were performed in triplicate with varying concentrations of histidine, and the results of the three trials were averaged to obtain kinetic constants. *F*, Michaelis-Menten plot for *F. nucleatum* ATCC 23726 histidine racemase enzyme with D-histidine.

Table S2**.** Kinetic parameters of *F. nucleatum* ATCC 23726 HisR with histidine as a substrate.

|  | *K*_M_ | *k_c_*_at_ | *k*_cat_/K_M_ |
| --- | --- | --- | --- |
| L-Histidine | 5 mM | 42 s^-1^ | 9 × 10^3^ M^-1^s^-1^ |
| D-Histidine | 18 mM | 64 s^-1^ | 4 × 10^3^ M^-1^s^-1^ |


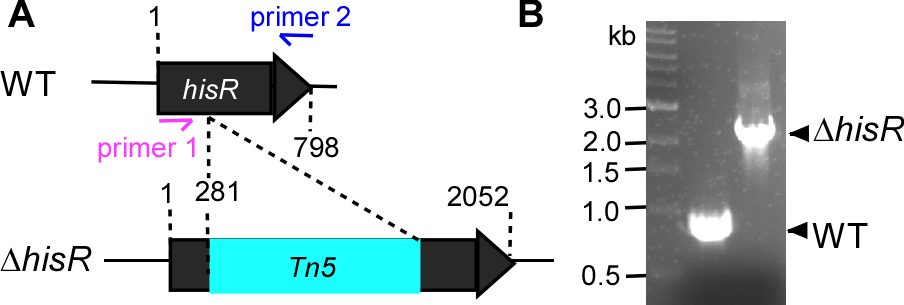


Figure S10**. Generation of *ΔhisR* strain of *F. nucleatum* ATCC 23726 using Tn*5* transposon mutagenesis.** *A*, Construction of *ΔhisR* strain of *F. nucleatum* ATCC 23726 (*ΔhisR*) using Tn*5* transposon system. *ΔhisR* strains are resistant to thiamphenicol. *B*, PCR confirmation of insertion of *Tn5* into *hisR*. The wild-type organism produces a PCR product of 798 bp, while the mutant produces a product of 2053 bp.

# Supporting Information Methods

# Protein and DNA Sequences

| **Protein** | **Sequence** | **MW (Da)** | **pI** | **Extinction coefficient (reduced thiols)** |
| --- | --- | --- | --- | --- |
| *F. nucleatum* ATCC 25586 HisR | MDRKVQVLDFIKINPAGNITILIDNFDIYDKNIPKLSEEIMKETNLYAEQVGFIKDSHLQMMGGEFCGNASRAFASLLAFRDKDFSKQKNYNITCSGESKVLDVDVRNDGAKNKFLAKIKMPKFLSLEEINVDEYKLGLVRFSGINHFIFNIKENKETSFENIIDLVKKYLSNEEYSAFGIMFFDSDNLSMKPYVYVKEVGSGVYENSCASGTTALGYYLKKCKNLDRAKIVQPNGWLEYIIENDEMYIDGPVEIIAEGKIYIGK**HHHHHH** | 31062 | 5.94 | 26360 |
| *F. nucleatum* ATCC 23726 HisR | MDGKVQVLDFIKINPAGNITILIDNFDIYDKNIPKLSEEIMKETNLYAEQVGFIKEKHLQMMGGEFCGNASRSFASLLAFRDKDFSEQKNYSITCSGESEVLDVDVRTDGAKNKFLAKIKMPKFISLEEISIDEYKLGLVRFSGISHFIFNIKENKETSFENIIDLVKKYLSNEDYSAFGIMFFDKDNLSMKPYVYVKELESGIYENSCASGTTALGYYLKKYKNLDRAKVVQPNGWLEYIIENDEMYIDGPVEIVAEGKVYI**HHHHHH** | 30915 | 5.35 | 27850 |

***Fusobacterium nucleatum* ATCC 25586 HisR codon optimized DNA sequence inserted into pET-24b(+) at 5’ *Nde*I and 3’ *Hind*III restriction sites:**

CAT**ATGGATCGCAAGGTGCAAGTGTTGGATTTTATTAAAATCAATCCGGCAGGAAATATCACCATTTTGATCGACAACTTTGACATATATGATAAGAACATTCCGAAACTGTCTGAGGAGATTATGAAAGAGACCAATCTTTATGCTGAGCAGGTAGGGTTCATCAAAGACTCGCATCTGCAAATGATGGGAGGGGAATTTTGTGGCAATGCTTCCAGAGCCTTTGCCTCtTAGCGTTTCGCGACAAGGACTTTTCGAAACAAAAGAACTATAACATAACGTGCTCTGGAGAATCCAAGGTTCTTGACGTTGACGTTCGGAATGACGGAGCGAAGAACAAATTCTTAGCGAAGATCAAAATGCCAAAGTTTCTTTCGCTGGAAGAAATTAATGTAGACGAGTACAAGTTGGGGTTAGTGCGCTTCAGTGGGATAAACCATTTCATCTTCAACATAAAGGAAAATAAAGAGACATCATTCGAGAATATAATAGACTTGGTAAAGAAATACTTATCCAACGAAGAATACAGCGCTTTTGGTATAATGTTTTTCGACTCAGATAATCTTTCAATGAAGCCTTACGTGTATGTCAAGGAAGTTGGGTCTGGAGTGTATGAGAATAGTTGTGCGTCAGGTACGACAGCGCTTGGTTATTATCTGAAGAAGTGTAAGAATTTGGATAGAGCTAAAATAGTTCAGCCGAATGGATGGCTTGAGTACATTATCGAAAACGACGAGATGTATATTGACGGGCCTGTCGAAATCATTGCAGAGGGCAAAATCTATATCGGTAAGCATCACCACCATCACCACTAA**GCTT

***Fusobacterium nucleatum* ATCC 23726 HisR codon optimized DNA sequence inserted into pET-24b(+) at 5’ *Nde*I and 3’ *Hind*III restriction sites:**

CAT**ATGGACGGAAAGGTACAAGTGCTTGATTTCATCAAGATTAACCCCGCAGGCAACATCACTATCCTTATCGATAACTTTGATATTTACGACAAGAACATCCCCAAGCTTTCGGAGGAAATTATGAAAGAGACTAATTTGTATGCTGAGCAAGTAGGCTTTATCAAAGAGAAGCATTTGCAGATGATGGGTGGGGAGTTTTGCGGAAACGCTTCACGTTCCTTTGCATCTCTTCTGGCCTTCCGCGATAAAGACTTCTCTGAACAAAAGAACTATAGCATCACATGTTCCGGGGAATCTGAGGTTTTGGACGTAGATGTTCGCACAGATGGTGCTAAGAATAAATTTCTTGCTAAAATTAAAATGCCAAAGTTTATCTCATTGGAAGAAATCTCCATCGATGAGTATAAACTTGGCCTTGTTCGCTTTTCTGGTATTAGCCATTTCATCTTTAACATTAAGGAGAACAAGGAGACGAGCTTCGAGAACATTATTGATCTTGTCAAGAAGTACCTGAGTAATGAGGACTACTCCGCCTTCGGGATTATGTTTTTTGACAAGGACAATTTGAGTATGAAACCGTATGTCTATGTAAAAGAATTAGAATCAGGCATTTACGAGAATTCGTGCGCCTCTGGAACTACCGCCCTTGGGTACTACCTTAAGAAATATAAAAACTTGGACCGCGCGAAAGTAGTCCAGCCTAATGGATGGTTAGAATATATCATCGAGAACGACGAAATGTACATCGACGGTCCCGTCGAAATTGTGGCCGAAGGGAAAGTATATATTCACCATCATCATCATCACTAA**GCTT

**C67S HisR mutant DNA sequence from *Fusobacterium nucleatum* ATCC 25586 inserted into pET-24b(+) at 5’ *Nde*I and 3’ *Hind*III restriction sites:**

CAT**ATGGATCGCAAGGTGCAAGTGTTGGATTTTATTAAAATCAATCCGGCAGGAAATATCACCATTTTGATCGACAACTTTGACATATATGATAAGAACATTCCGAAACTGTCTGAGGAGATTATGAAAGAGACCAATCTTTATGCTGAGCAGGTAGGGTTCATCAAAGACTCGCATCTGCAAATGATGGGAGGGGAATTTTCTGGCAATGCTTCCAGAGCCTTTGCCTCCTTGTTAGCGTTTCGCGACAAGGACTTTTCGAAACAAAAGAACTATAACATAACGTGCTCTGGAGAATCCAAGGTTCTTGACGTTGACGTTCGGAATGACGGAGCGAAGAACAAATTCTTAGCGAAGATCAAAATGCCAAAGTTTCTTTCGCTGGAAGAAATTAATGTAGACGAGTACAAGTTGGGGTTAGTGCGCTTCAGTGGGATAAACCATTTCATCTTCAACATAAAGGAAAATAAAGAGACATCATTCGAGAATATAATAGACTTGGTAAAGAAATACTTATCCAACGAAGAATACAGCGCTTTTGGTATAATGTTTTTCGACTCAGATAATCTTTCAATGAAGCCTTACGTGTATGTCAAGGAAGTTGGGTCTGGAGTGTATGAGAATAGTTGTGCGTCAGGTACGACAGCGCTTGGTTATTATCTGAAGAAGTGTAAGAATTTGGATAGAGCTAAAATAGTTCAGCCGAATGGATGGCTTGAGTACATTATCGAAAACGACGAGATGTATATTGACGGGCCTGTCGAAATCATTGCAGAGGGCAAAATCTATATCGGTAAGCATCACCACCATCACCACTAA**GCTT

**C67S HisR mutant DNA sequence from *Fusobacterium nucleatum* ATCC 25586 inserted into pET-24b(+) at 5’ *Nde*I and 3’ *Hind*III restriction sites:**

CAT**ATGGATCGCAAGGTGCAAGTGTTGGATTTTATTAAAATCAATCCGGCAGGAAATATCACCATTTTGATCGACAACTTTGACATATATGATAAGAACATTCCGAAACTGTCTGAGGAGATTATGAAAGAGACCAATCTTTATGCTGAGCAGGTAGGGTTCATCAAAGACTCGCATCTGCAAATGATGGGAGGGGAATTTTGCGGCAATGCTTCCAGAGCCTTTGCCTCCTTGTTAGCGTTTCGCGACAAGGACTTTTCGAAACAAAAGAACTATAACATAACGTGCTCTGGAGAATCCAAGGTTCTTGACGTTGACGTTCGGAATGACGGAGCGAAGAACAAATTCTTAGCGAAGATCAAAATGCCAAAGTTTCTTTCGCTGGAAGAAATTAATGTAGACGAGTACAAGTTGGGGTTAGTGCGCTTCAGTGGGATAAACCATTTCATCTTCAACATAAAGGAAAATAAAGAGACATCATTCGAGAATATAATAGACTTGGTAAAGAAATACTTATCCAACGAAGAATACAGCGCTTTTGGTATAATGTTTTTCGACTCAGATAATCTTTCAATGAAGCCTTACGTGTATGTCAAGGAAGTTGGGTCTGGAGTGTATGAGAATAGTTCTGCGTCAGGTACGACAGCGCTTGGTTATTATCTGAAGAAGTGTAAGAATTTGGATAGAGCTAAAATAGTTCAGCCGAATGGATGGCTTGAGTACATTATCGAAAACGACGAGATGTATATTGACGGGCCTGTCGAAATCATTGCAGAGGGCAAAATCTATATCGGTAAGCATCACCACCATCACCACTAA**GCTT

# Construction of Tn*5* Mutant in *F. nucleatum* ATCC 23726

**DNA sequence of *hisR* in wild-type in ATCC 23726:**

atgGATGGAAAAGTGCAAGTTTTAGATTTTATAAAAATTAATCCTGCTGGAAATATTACAATACTTATAGATAATTTTGATATTTATGATAAAAATATTCCAAAATTATCAGAAGAAATTATGAAAGAAACTAATCTCTATGCAGAACAAGTAGGATTTATTAAAGAAAAACACCTCCAAATGATGGGTGGAGAATTTTGTGGAAATGCAAGTAGGTCTTTTGCAAGTCTTTTAGCTTTTAGAGATAAAGATTTTTCTGAACAAAAAAATTATAGTATAACTTGTTCAGGTGAGAGTGAAGTTTTAGATGTAGATGTAAGAACTGATGGAGCTAAAAATAAGTTCTTAGCTAAAATTAAAATGCCTAAGTTTATAAGTCTTGAAGAAATTAGTATAGATGAATATAAATTAGGGCTTGTTAGATTTTCTGGAATAAGTCATTTTATTTTTAACATTAAGGAAAATAAAGAAACTAGTTTTGAAAATATTATAGATTTAGTTAAAAAATATCTATCTAATGAAGATTATTCAGCCTTTGGAATTATGTTCTTTGATAAAGATAACTTATCAATGAAACCTTATGTCTATGTAAAAGAACTTGAAAGTGGAATATATGAAAATAGCTGTGCTTCAGGAACAACTGCATTGGGCTATTATTTAAAGAAATATAAAAATTTAGACAGAGCTAAGGTTGTTCAGCCTAATGGTTGGTTAGAATATATTATTGAAAATGATGAAATGTATATAGATGGACCTGTTGAAATAGTAGCAGAAGGAAAGGTGTACATAtga

**DNA sequence of *ΔhisR* in ATCC 23726 (Tn*5* highlighted in green):**

atgGATGGAAAAGTGCAAGTTTTAGATTTTATAAAAATTAATCCTGCTGGAAATATTACAATACTTATAGATAATTTTGATATTTATGATAAAAATATTCCAAAATTATCAGAAGAAATTATGAAAGAAACTAATCTCTATGCAGAACAAGTAGGATTTATTAAAGAAAAACACCTCCAAATGATGGGTGGAGAATTTTGTGGAAATGCAAGTAGGTCTTTTGCAAGTCTTTTAGCTTTTAGAGATAAAGATTTTTCTGAACAAAAAAATTATAGTATAACCTGTCTCTTATACACATCTCAACCATCATCGATGAATTTTCTCGGGTGTTCTCGCATATTGGCTCGAATTCAACGAGTGAAAAAGTACTGGTCCCTAGCGCCTACGGGGAATTTGTATCGATAAGGGGTACAAATTCCCACTAAGCGCTCGGCGGGGATCGATCCCGGGTACGTACCCGGCAGTTTTTCTTTTTCGGCAAGTGTTCAAGAAGTTATTAAGTCGGGAGTGCAGTCGAAGTGGGCAAGTTGAAAAATTCACAAAAATGTGGTATAATATCTTTGTTCATTAGAGCGATAAACTTGAATTTGAGAGGGAACTTAGATGGTATTTGAAAAAATTGATAAAAATAGTTGGAACAGAAAAGAGTATTTTGACCACTACTTTGCAAGTGTACCTTGTACATACAGCATGACCGTTAAAGTGGATATCACACAAATAAAGGAAAAGGGAATGAAACTATATCCTGCAATGCTTTATTATATTGCAATGATTGTAAACCGCCATTCAGAGTTTAGGACGGCAATCAATCAAGATGGTGAATTGGGGATATATGATGAGATGATACCAAGCTATACAATATTTCACAATGATACTGAAACATTTTCCAGCCTTTGGACTGAGTGTAAGTCTGACTTTAAATCATTTTTAGCAGATTATGAAAGTGATACGCAACGGTATGGAAACAATCATAGAATGGAAGGAAAGCCAAATGCTCCGGAAAACATTTTTAATGTATCTATGATACCGTGGTCAACCTTCGATGGCTTTAATCTGAATTTGCAGAAAGGATATGATTATTTGATTCCTATTTTTACTATGGGGAAATATTATAAAGAAGATAACAAAATTATACTTCCTTTGGCAATTCAAGTTCATCACGCAGTATGTGACGGATTTCACATTTGCCGTTTTGTAAACGAATTGCAGGAATTGATAAATAGTTAACTTCAGGTTTGTCTGTAACTAAAAACAAGTATTTAAGCAAAAACATCGTAGAAATACGGTGTTTTTTGTTACCCTAAAATCTACAATTTTATACATAACCACAGGTTAGTACAAAGACCTTGTGTTTCTTTTTGAAAGGCTTAAAACAAGGATTTTTCCTTGATTTAAGCCCCGAAAAGCAACACAACCAAGGTTTTAGAATTCGAGCTCGGTACCCGGGGATCCTCTAGAGTCGACCTGCAGGCATGCAAGCTTGCCAACGACTACGCACTAGCCAACAAGAGCTTCAGGGTTGAGATGTGTATAAGAGACAGTTGTTCAGGTGAGAGTGAAGTTTTAGATGTAGATGTAAGAACTGATGGAGCTAAAAATAAGTTCTTAGCTAAAATTAAAATGCCTAAGTTTATAAGTCTTGAAGAAATTAGTATAGATGAATATAAATTAGGGCTTGTTAGATTTTCTGGAATAAGTCATTTTATTTTTAACATTAAGGAAAATAAAGAAACTAGTTTTGAAAATATTATAGATTTAGTTAAAAAATATCTATCTAATGAAGATTATTCAGCCTTTGGAATTATGTTCTTTGATAAAGATAACTTATCAATGAAACCTTATGTCTATGTAAAAGAACTTGAAAGTGGAATATATGAAAATAGCTGTGCTTCAGGAACAACTGCATTGGGCTATTATTTAAAGAAATATAAAAATTTAGACAGAGCTAAGGTTGTTCAGCCTAATGGTTGGTTAGAATATATTATTGAAAATGATGAAATGTATATAGATGGACCTGTTGAAATAGTAGCAGAAGGAAAGGTGTACATAtga

**PCR Primers**

(primer 1) ATGGATGGAAAAGTGCAAGTTTTAGAT wild type 798 bp

(primer 2) TCATATGTACACCTTTCCTTCTGCT mutant 2000 bp

# Synthesis of Oxazole Analogue of Histidine


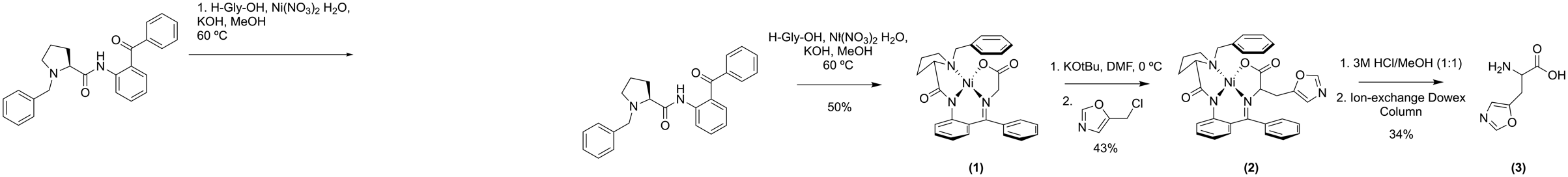


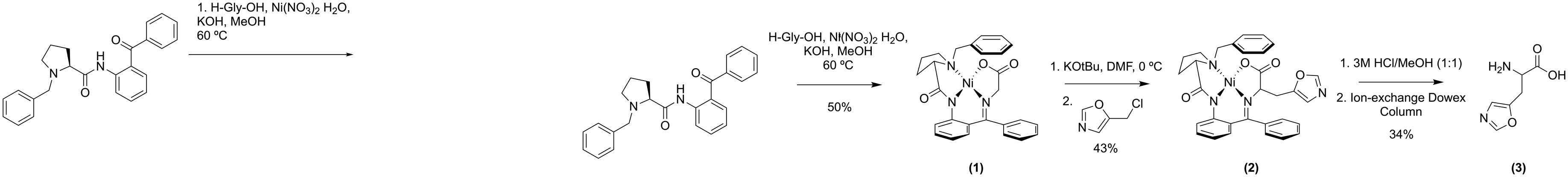


This known compound was synthesized based on a literature procedure (1). (*S*)-*N* -(2-Benzoylphenyl)-1-benzylpyrrolidine-2-carboxamide (1.0 g, 2.6 mmol, 1.0 equiv), Ni(NO_3_)· 6H_2_O (30.2 g, 5.1 mmol, 2.0 equiv), and L-glycine (0.97 g, 13.0 mmol, 5.0 equiv) was dissolved in 15 mL of dry methanol under argon at 45 °C. Freshly ground KOH (1.0 g, 18.1 mmol, 7.0 equiv) was added to the reaction at 45 °C and stirred for 1 h, then stirred for 1 h at 60 °C. The dark red reaction was cooled to 0 °C and acetic acid (1.0 mL) was added to quench residual KOH. The reaction was then concentrated *in vacuo*, resuspended in H_2_O, and the crude product was collected by vacuum filtration. The solid was then purified by column chromatography using an elution of gradient 5 – 15 % MeOH in EtOAc yielding a red solid (0.64 g, 1.3 mmol, 50%); R_f_ 0.3 on SiO_2_, 9:1 EtOAc:MeOH. The product exhibited spectra data identical to previous reports.


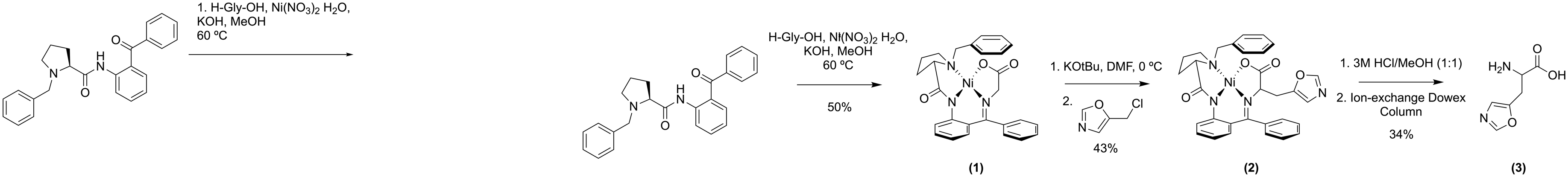


This compound was synthesized based on a literature procedure (2). A solution of Nickel-Schiff complex compound (2.0 g, 4.0 mmol) was dissolved in anhydrous DMF (40 mL) and cooled to 0 °C. To the solution was added KOtBu (0.7 g, 6.2 mmol) followed by 5-(chloromethyl)oxazole (0.53 g, 4.5 mmol) in anhydrous DMF (2.0 mL). The reaction mixture was left to stir for 45 mins at 0 °C, and then brought up to room temperature and stirred for an additional 16 hr. The reaction was quenched using 4 mL of 5 % aqueous acetic acid and the crude mixture was concentrated *in vacuo*. The solid was resuspended in water and extracted with DCM (3 x 10 mL). The organic layers were pooled, washed with brine, dried over MgSO_4_, and concentrated *in vacuo*. The crude product was purified with gradient elution flash column chromatography (2.5%—15% MeOH in EtOAc) to afford **(2)** as a red solid, R*_f_* = 0.4 (20% MeOH in EtOAc), (1.0 g, 43%). ^1^H-NMR (700 MHz, CD_3_OD) δH 8.35—8.31 (3H, m), 7.97 (d, 2H, *J* = 8.0 Hz), 7.66 (td, 1H, *J* = 7.5, 1.0 Hz), 7.62 (tt, 1H, *J* = 9.0, 7.0, 2.0 Hz), 7.57—7.54 (m, 1H) 7.49—7.48 (m, 1H), 7.35—7.31 (m, 2H), 7.5 (s, 1H,), 7.11—7.14 (m, 3H), 6.72 (ddd, 1H, *J* = 8.0, 6.8, 1.2 Hz), 6.68 (dd, 1H, *J* = 8.0, 2.0 Hz), 4.25 (dd, 1H, *J* = 6.9, 3.4 Hz), 4.11 (d, 1H, *J* = 12.6 Hz), 3.53 (dd, 1H, *J* = 10.6, 6.6 Hz), 3.49 (d, 1H, *J* = 12.5 Hz), 3.26 (ddd, 1H, *J* = 10.2, 6.6, 2.7 Hz), 3.11 (dd, 1H, *J* = 15.4, 3.4 Hz), 2.98 (dd, 1H, *J* = 15.4, 6.9 Hz), 2.87 (ddd, 1H, *J*=10.6, 5.2, 3.1 Hz), 2.58 – 2.47 (m, 1H), 2.42 (ddt, 1H, *J* = 13.1, 6.6, 3.4 Hz), 2.17 (td, 1H, *J* = 10.9, 6.4 Hz) 2.07 (m, 1H);^13^C-NMR (176 MHz, CD_3_OD) δ 182.7, 181.2, 174.3, 153.9, 149.3, 136.3, 134.7, 133.4, 132.6, 131.3, 130.5, 130.2, 130.0, 129.9, 129.5, 129.3, 128.3, 127.7, 125.9, 124.8, 122.2, 72.6, 70.2, 67.8, 65.4, 59.3, 53.0, 31.8, 30.1, 24.5, 19.7; HRMS (ESI) Calcd for C_31_H_29_N_4_NiO_4_ [M+H]^+^ 579.1537, found 579.1538


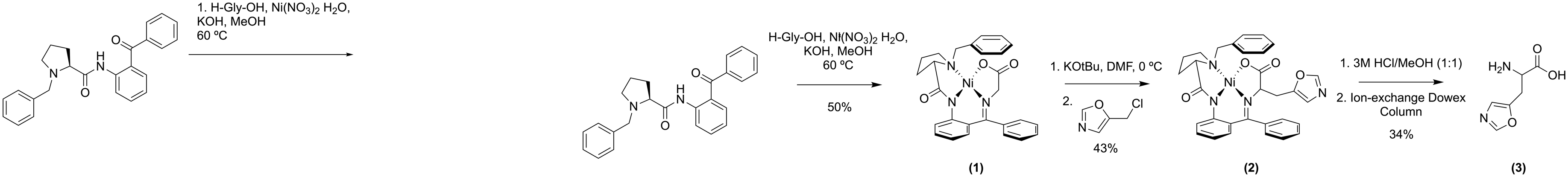


This known compound was synthesized based on literature procedure (3). The Ni(II)- alkylated product **(2)** (152 mg, 0.26 mmol) in MeOH (0.5 mL) was added dropwise to a solution of 1:1 3 M HCl: MeOH (2 mL) at 65 °C and was stirred for 1 hr. The reaction mixture was concentrated *in vacuo* and the residue was dissolved in water (1 mL) and evaporated. NH_4_OH (1mL), and then water (1 mL) was added and the mixture was concentrated *in vacuo*. The residue was then re-dissolved in water (1 mL) and CHCl_3_ (1 mL). The organic phase was separated, and the aqueous phase was washed with CHCl_3_. The aqueous layer was then loaded into a pre-equilibrated ion-exchange column (DOWEX 50WX8, 100-200 mesh resin) and the amino acid was eluted using H_2_O:NH_4_OH (10—80% gradient) yielding **(3**) (13 mg, 34%) as a white solid. Exhibited spectra data identical to previous reports.^3^

# Supporting Information References

1. Belokon’, Y. N., Tararov, V. I., Maleev, V. I., Savel’eva, T. F., and Ryzhov, M. G. (1998) Improved procedures for the synthesis of (S)-2-[N-(N′-benzylprolyl)amino]benzophenone (BPB) and Ni(II) complexes of Schiff’s bases derived from BPB and amino acids. *Tetrahedron Asymmetry*. **9**, 4249–4252

2. Vuong, W., Mosquera-Guagua, F., Sanichar, R., McDonald, T. R., Ernst, O. P., Wang, L., and Vederas, J. C. (2019) Synthesis of Chiral Spin-Labeled Amino Acids. *Org. Lett*. **21**, 10149–10153

3. Okada, N., Kyosuke, U., Masatoshi, T., Shunichi, N., Kotaro, T., Ashizawa, T., *et al*. (2023) Novel Amino Acid Derivatives. U.S. Patent US2023257343A1, August 17, 2023.
